# Cyber loss model risk translates to premium mispricing and risk sensitivity

**DOI:** 10.1057/s41288-023-00285-x

**Published:** 2023-03-18

**Authors:** Gareth W. Peters, Matteo Malavasi, Georgy Sofronov, Pavel V. Shevchenko, Stefan Trück, Jiwook Jang

**Affiliations:** 1 Statistics & Applied Probability, University of California Santa Barbara, Santa Barbara, USA; 2 Actuarial Studies and Business Analytics, Macquarie University, Macquarie Park, Australia; 3 Mathematical and Physical Sciences, Macquarie University, Macquarie Park, Australia; 4 Center for Econometrics and Business Analytics, Saint-Petersburg State University, St Petersburg, Russia

**Keywords:** Cyber risk, Cyber insurance, Model risk, Risk sensitivity, Robust estimation, Robust dependence estimation

## Abstract

In this paper we focus on model risk and risk sensitivity when addressing the insurability of cyber risk. The standard statistical approaches to assessment of insurability and potential mispricing are enhanced in several aspects involving consideration of model risk. Model risk can arise from model uncertainty and parameter uncertainty. We demonstrate how to quantify the effect of model risk in this analysis by incorporating various robust estimators for key model parameters that apply in both marginal and joint cyber risk loss process modelling. Through this analysis we are able to address the question that, to the best of our knowledge, no other study has investigated in the context of cyber risk: is model risk present in cyber risk data, and how does is it translate into premium mispricing? We believe our findings should complement existing studies seeking to explore the insurability of cyber losses.

## Introduction

Cyber risk continues to gain relevance in our society, as companies and enterprises increasingly rely on information systems. For a detailed overview of the state of cyber risk understanding in insurance contexts, see Eling (2020). A successful cyberattack can cause major damage to a public or private institution. It can directly affect the budgetary bottom line, in addition to a business’ standing and consumer trust. Cybersecurity breaches can be categorised basically into three depending on the types of losses they cause following the attack: financial, reputational damage and legal, see detailed discussions in Eling and Schnell (2016) and Peters et al. (2018).

Furthermore, cyberattacks can carry a significant direct economic and financial cost, see discussions in Romanosky et al. (2017) and the empirical analysis in Biener et al. (2015), Edwards et al. (2016) and Shevchenko et al. (2021). These costs can manifest for instance as losses due to: theft of corporate information; theft of financial information such as customer records; direct theft of money or assets; business disruption of critical systems such as trading or transaction processing; and the loss of business or contracts, to name but a few (see Peters et al. 2018 for a cluster analysis of cyber event types for insurance and risk contexts). Furthermore, there are also often significant losses arising from incurred costs associated with repairing affected systems, networks and devices. This is often required after major events in order to meet regulatory standards or satisfy investors or clients of the risk reduction changes made post significant cyber events, see an overview on discussions on Basel banking regulation requirements for operational risk cyber loss in Cruz et al. (2015).

After a cyberattack there are also a variety of indirect costs that often arise which may be due to reputational damage borne from news of the attack reaching the public or customers affected by data breach of their records, see discussions on data breach fines in cyber risk in Ceross and Simpson (2017). Cyberattacks can damage a business’ reputation and erode the trust of the customer, leading to customer attrition. The effect of reputational damage can even impact on an institution’s suppliers, or affect relationships with partners, investors and other third parties vested in a business. Other impacts from cyber events can include legal and regulatory consequences. In many jurisdictions, both private and public entities are required to provide certain guarantees on data privacy under data protection and privacy laws which require firms to manage the security of all personal data being held on staff and customers. If this data is accidentally or deliberately compromised, and the firm in question can be deemed to have failed to deploy appropriate security measures, they may face fines and regulatory sanctions from multiple jurisdictions.

As such, it is increasingly becoming apparent that mitigation of cyber risk and cyber losses alone will not suffice to protect both public and private institutions from the potential for catastrophic monetary losses arising from cyberattacks. Therefore, upon the realisation that cyberattacks can never truly be completely mitigated, especially with the increasing pace of technology adoption and growth, then there is a growing need to find effective risk transfer strategies. One such strategy, not to mitigate a cyber loss but rather to ensure that the affected institution or firm is able to recover and fund any required losses, is through insurance and reinsurance markets. Cyber risk insurance markets and available products are still very much in their infancy, see discussions in Eling and Schnell (2016) and Eling (2018) and a recent U.S. case study in Xie et al. (2020). As the interest in the effects of cyber risk grows, so does the number of actuarial studies tackling several important questions on cyber risk (Eling and Loperfido 2017; Eling and Wirfs 2019; Edwards et al. 2016; Jung 2021).

In this manuscript, the focus is on aspects of the perception of the insurability of cyber risk. Two factors make this work distinct from previous studies of a related nature: the first is the fact that we have used one of the industry gold standards for cyber loss data, Advisen Cyber Loss Data (https://www.advisenltd.com/data/cyber-loss-data), which represents a comprehensive data set on cyber monetary losses, from which we form our analysis and conclusions; the second, perhaps more important aspect is that we question the standard statistical approaches to assess the question of insurability in several important aspects. In particular, one may conclude that we assess the question of insurability of cyber loss taking into account a previously unaccounted for dimension related to model risk. We seek to answer a question that, to the best of our knowledge, no other study has investigated in the context of cyber risk: is model risk present in cyber risk data, and how does is it translate into premium mispricing?

Model risk can arise from two different factors: model uncertainty and parameter uncertainty. While model uncertainty generally refers to the assumptions that one makes in developing a statistical model representation, parameter uncertainty revolves around the idea of predictive inference (Fröhlich and Weng 2018). In this paper we focus on both aspects of model structure uncertainty as well as parameter uncertainty, and investigate two main channels of transmissions, using the Advisen cyber loss dataset. In this regard we focussed on two core components related to assessing the question of insurability for both an individual cyber risk threat type or a portfolio of multiple cyber risk threat types. The first component is the marginal tail behaviour of a cyber risk loss process and how assumptions regarding the validity of core details in reported losses and the completeness of such records, obtained from Freedom of Information (FOI) requests, may influence the outcome of determinations of the insurability of cyber losses. We achieve this by considering a variety of parametric and non-parametric estimators for the tail index of the loss processes under study and we add to this analysis the dimension of robust estimation, which involves the ability to question the validity, completeness, quantisation, providence, accuracy and veracity of losses by trimming or weighting exceptionally large losses that directly influence the marginal question of insurability. Secondly, we also study the effect of uncertainty in the model structure and estimation of dependence in cyber risk losses. To achieve this we employ novel methods for the quantification of copula dependence structures, robust estimation techniques for correlation analysis and tail dependence estimation. This aspect of model risk allows us to assess the impact on diversification of cyber risk that may or may not be present in an insurer’s portfolio, should they offer cyber risk products to clients across a range of different cyber risk loss types or over a range of different industry sectors.

### Contributions and outline

Our studies are undertaken in two parts and offer a variety of contributions to the understanding of model risk, parameter uncertainty and its translation to premium mispricing in cyber risk settings. Ultimately, we argue this provides a new dimension to understanding the insurability of cyber risk as quantified by required insurance premiums.

First, we study model and parameter uncertainty risk as it relates to the key idea of tail index estimation. In particular we demonstrate significant challenges with working with cyber loss data when estimating tail indexes and we demonstrate the variation obtained in the different parametric and non-parametric tail index estimators under varying assumptions on the data quality and accuracy as reflected in modifications to our estimators. As such, we first consider how parameter uncertainty impacts insurance premium calculations. Several studies have shown that cyber risk severity follows a heavy-tailed distribution (Eling and Wirfs 2019; Eling and Loperfido 2017; Edwards et al. 2016). We focus our analysis on the tail index of the cyber event severity distribution and recall some well-known facts about various estimators proposed by the literature.

Then, we aggregate cyber-related losses by business sector, adopting the North America Industry Classification (NAIC) which is the standard used by Federal statistical agencies in classifying business establishments for the purpose of collecting, analysing, and publishing statistical data related to the U.S. business economy. This categorisation was developed under the auspices of the Office of Management and Budget (OMB), and adopted in 1997 to replace the Standard Industrial Classification (SIC) system. It was developed jointly by the U.S. Economic Classification Policy Committee (ECPC), Statistics Canada, and Mexico’s Instituto Nacional de Estadistica y Geografia, to allow for a high level of comparability in business statistics among the North American countries. It is utilised by Advisen in their data collection categorisation and is provided for every loss event recorded. Based on loss data aggregated by NAIC, we then proceed to analyse and compare the tail index estimates using various estimators. We observe significant variations among tail index estimates, indicating the presence of parameter uncertainty. This acts as a motivation for robust and trimmed tail index estimators. In this process, we further explain how and why cyber loss data may need to be trimmed and then show the effect this has on the tail index estimations. Trimmed tail index estimators are a valid alternative in the context of cyber risk, where many extreme loss events are often of very high monetary amounts and one could question whether they do turn into realised losses, see Brazauskas and Serfling (2000), Zou et al. (2020), Goegebeur et al. (2014), Peng and Welsh (2001). Using the trimmed estimator in Bhattacharya et al. (2017), for different trimming values, we confirm once more the presence of parameter uncertainty given the great variability in the values of the tail index estimates. Ultimately, based on this estimation analysis, we then demonstrate for each tail index estimation method and set of assumptions, using both trimmed and non-trimmed estimators, how a utility-based pricing of an insurance premium, under an indifference pricing framework, produces variation/sensitivity for a single insurance line by NAIC. Ultimately, we demonstrate, using the zero utility principle, how parameter uncertainty translates into insurance premium mispricing risk, which could jeopardise the insurability of cyber risk with regard to required premiums to be incurred. We note that at present, premiums for such products are generally deemed prohibitive unless contracts are specifically designed to be bespoke and restricted in scope of coverage.

Next, we assess model and parameter uncertainty from a multivariate perspective, and determine how such model risk factors affect the diversification of an insurer’s portfolio of insured cyber risk lines, when multiple lines or multiple industry sectors are being offered coverage. In particular, we explore copula model uncertainty as well as robust versus non-robust estimators of correlation dependence. To achieve this we adopt a novel approach to estimate the incurred premium for a portfolio of loss types or business lines. In particular, we assess model risk as it relates to the key idea of diversification of risk from risk pooling. To achieve this we study the linear correlation for all NAICs, then we focus on the top five NAICs and study robust correlation analysis using three robust estimators: median-based Sum-of-Squares (SSD), Quadrant (sign) correlation coefficient and Minimum Covariance Determination (MCD). We are able to then demonstrate how the risk-based diversification coefficient will vary for differing robust estimators and the role this sensitivity will have on portfolio net coverage, thereby demonstrating sensitivity to premium calculations in marginal versus conditional risk profile perspectives. Lastly, we also fit a variety of pairwise copulas on quarterly data and show sensitivity to these fits and the resulting sensitivity of premiums calculated.

The manuscript is structured as follows. The “Significance of cyber risk losses and cyber insurance markets” section presents an overview of the significance of cyber losses and a brief discussion on cyber insurance markets. In the “Data description and attributes” section a detailed data description is provided for the Advisen cyber loss data set, followed by several sets of empirical data analysis of the cyber risk data according to Advisen risk types and industry sectors determined by the NAIC codes. The “Quantifying heavy tails in cyber risk loss models” section addresses the quantification of heavy tails in cyber risk losses. A variety of methods are explored including smoothed Hill plots, extremogram estimators and analysis of tail index estimators from a variety of different statistical perspectives, including empirical characteristic function asymptotic regression methods, Hill type estimation methods and variations. These methods are briefly mathematically outlined and then applied to study cyber risk loss data from Advisen. The “Robust trimmed hill estimators for cyber losses” section outlines the challenges associated with working with real world cyber loss data that include: inaccuracies, rounding, truncation, partial settlement and unreliable massive reported cyber total losses. To address this and determine how it can manifest as a form of model risk via model uncertainty, and parameters uncertainty, the “Dealing with real world cyber data: inaccurate, rounded, truncated, partially settled unreliable massive reported cyber total losses” and “Dependence and tail behaviour estimation on Advisen NAIC cyber losses” sections introduce robust methods. This includes overviews of relevant classes of robust trimmed Hill estimators that are applied subsequently to the cyber loss data. The “Dependence and tail behaviour estimation on Advisen NAIC cyber losses” section presents a comprehensive analysis of the Advisen Loss Data using the various proposed robust tail index estimation methods as well as a study of robust dependence. Subsequently, a detailed insurance pricing example is performed for various cyber insurance lines of business to show how model risk in tail index estimation transfers to potential for mispricing in cyber risk insurance, ranging from uninsurable due to exorbitant costs through to affordable, depending on the modelling approach adopted. Furthermore, a detailed analysis of dependence between different cyber risk loss processes is studied via robust dependence estimation methods and copula estimation methods and a little-known Monte Carlo−based simulation method is detailed in order to perform insurance pricing and portfolio diversification assessment in standard and robust contexts to further assess aspects of multivariate model risk in insurance pricing contexts. The paper concludes with the “Conclusions” section.

## Significance of cyber risk losses and cyber insurance markets

We begin this section with a brief overview of the significance of cyber losses and then we provide a brief discussion on cyber insurance markets. Comprehensive and detailed discussions can be found in Eling and Schnell (2016), Peters et al. (2018), Falco et al. (2019), Eling (2018) and the challenges with quantitative analysis in this multidisciplinary domain are discussed in Falco et al. (2019).

### Global significance of cyber risk losses

According to a recent estimate provided in the Global Risk Report by the World Economic Forum (2020a), losses from cyber-related risks are expected to increase by up to USD 6 trillion 2021. Recently, McShane et al. (2021) noted that Hiscox insurance stated that in 2020, the median cost of a cyberattack on a business increased from USD 10,000 in 2019 to USD 57,000 in 2020. These increases are not unexpected when one factors in the increasing digitisation of business and economic activities via the Internet of Things (IoT), cloud computing, mobile, blockchain and other innovative technologies. Financial losses from malicious cyber activities result from IT security/data/digital asset recovery, liability with respect to identity theft and data breaches, reputation/brand damage, legal liability, cyber extortion, regulatory defence, penalty coverage and business interruption. In the financial sector, cyber risk is classified by the Basel Committee on Banking Supervision (2006) as a category of operational risk, for instance affecting information and technology assets that can have consequences for the confidentiality, availability, and integrity of information and information systems (Cebula and Young 2010).

Not only are the losses substantial for given cyber risk events, but of concern is the fact that the frequency of malicious cyber activities is rapidly increasing, with the scope and nature dependent on an organisation’s industry, size and location. According to the Allianz Global Risk Barometer 2021 (Allianz 2021), cyber incidents (including cybercrime, IT failure/outage, data breaches, fines and penalties) are currently a top-three global business risk. It is therefore critical that corporations and governments focus on IT and network security enhancement. Unless public and private sector organisations have effective cybersecurity plans and strategies in place, and tools to manage and mitigate losses from cyber risks, cyber events have the potential to affect their business significantly, possibly damaging hard-earned reputations irreparably (McShane et al. 2021).

Furthermore, the impact of COVID-19 has driven business and economic activities at an accelerated rate into cyberspace, which could significantly increase the frequency and impact of cyber events around the globe, with alarming consequences for public and private sector organisations (Lallie et al. 2021).

The lack of historical data on losses from cyber risk is another challenge to model the frequency and severity of individual cyber-related events (Biener et al. 2015; Eling and Wirfs 2019; Gordon et al. 2003; World Economic Forum 2020b). For example, in Australia, it only became mandatory for breached organisations to notify their data breach details in February 2018 (see Parliament of Australia 2017). Many countries around the world are in a similar situation, such that often only very limited data on losses from cyber-related events is available. This makes the design of adequate models for the quantification of cyber risks very difficult.

**Fig. 1:**
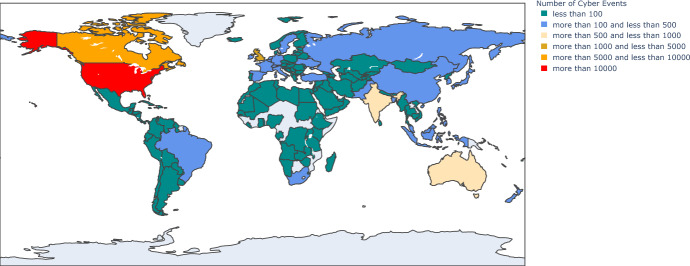
Number of cyber events by country during the period 2008–2020 across all loss categories in the Advisen cyber risk data set

The Advisen data set utilised in this manuscript is considered one of the current industry gold standards for cyber risk data collection; we will discuss in greater detail this data set in the “Data description and attributes” section. Figure 1 illustrates the number of events in the Advisen database by country; the studies undertaken in this manuscript focus on the U.S. but we briefly outline the global perspective from this data set. The vast majority of the recorded events in this database occurred in the U.S. (83.09%), while only a minority of events is recorded for the entire European Union (2.65%), Asia (3.17%) or Oceania (1.04%). As mentioned earlier, given the focus of the data set on the U.S. companies presented have been classified according to the NAIC system by Advisen.

In the following, we focus our analysis on non-zero losses in the dataset, i.e. 4,667 cyber events. Note that the share of these losses in the entire database is only 3.53% of the total events. However, given our emphasis on the severity of cyber-related events, we have to rely on events where information on the magnitude of the loss was provided.

Table 1 provides descriptive statistics of non-zero losses for each cyber risk category. We find substantial differences for the number of non-zero loss events across the different risk categories. While we observe over 1900 non-zero losses for the category *Privacy—Unauthorised Contact or Disclosure*, only six non-zero losses are observed for the category *Industrial Controls* throughout the sample period. We also find heterogeneity in the magnitude of losses across the different categories. All risk categories exhibit a mean loss that is higher than the median, indicating that the loss distribution is skewed to the right, potentially exhibiting heavy tails. In some cases this effect is so pronounced that the mean is more than 100 times higher than the median. Table 1 also illustrates that losses from cyber events typically have a very high standard deviation, positive skewness paired with high kurtosis. Overall, the descriptive statistics in Table 1 also seem to confirm earlier results on cyber-related losses typically following heavy-tailed distributions; see, e.g. Maillart and Sornette (2010), Edwards et al. (2016) and Eling and Loperfido (2017).

**Table 1:** This table reports descriptive statistics of cyber risk related losses aggregated by category

Risk category	N	Mean	Median	StDev	Skew	Kurt
Phishing, Spoofing, Social Engineering	202	12.36	0.57	79.3	9.72	95.53
Privacy—Unauthorised Contact or Disclosure	1916	3.05	0.03	23.8	31.75	1185.92
Data—Unintentional Disclosure	217	1.34	0.1	8.81	12.73	172.27
Privacy—Unauthorised Data Collection	133	46.77	0.45	434.07	11.18	124.5
Data—Malicious Breach	858	22.13	0.5	171.64	17.33	360.13
Identity—Fraudulent Use/Account Access	689	1.2	0.03	6.55	10.28	124.79
Data—Physically Lost or Stolen	97	23.91	0.24	202.14	9.63	91.16
Skimming & Physical Tampering	91	1.72	0.05	6.08	6.14	42.59
IT—Processing Errors	44	76.55	0.66	264.77	5.32	29.25
IT—Configuration/Implementation Errors	63	17.06	0.8	43.3	3.23	10.41
Network/Website Disruption	181	18.77	0.16	68.85	4.76	23.32
Cyber Extortion	137	0.52	0.01	2.78	6.86	48.24
Digital Breach/Identity Theft	11	469.22	30.0	1064.11	2.62	5.24
Denial of Service (DDOS)/System Disruption	1	0.39	0.39	–	–	–
Undetermined/Other	21	1.53	0.65	2.43	3.25	10.68
Industrial Controls and Operations	6	30.7	2.07	62.39	1.78	1.18

All dollar values are reported in million dollars. The losses exhibit great variability in terms of median and first four moments across the considered risk types. “Digital Breach/Identity Theft”, “IT—Processing Errors”, and “Privacy—Unauthorised Data Collection” have the highest average loss amongst all cyber risk categories

**Fig. 2:**
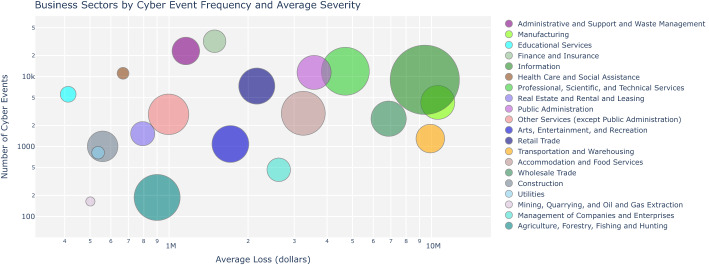
Frequency and severity of individual cyber-related events and the number of affected records (indicated by the size of the circle) across business sectors

Figure 2 shows the business sector ranked by frequency and severity of cyber events. Each circle represents a business sector, and its area corresponds to the average number of records affected by a cyber event, i.e. the larger the circle the more records have been affected.

Figure 2 also depicts the fact that monetary losses and the number of records affected vary across business sectors. Business sectors in the top right corner of the graph in Fig. 2 share some common features: they exhibit high average loss and high number of events, and a high average number of records affected (the bubbles have larger sizes than the sector in the top left corner of the graph). This seems to indicate that depending on the intrinsic nature of the business sector, for some sectors there is a connection between a high number of records stolen which translates into high losses. However, for other sectors, a larger number of records does not necessarily translate into greater losses.

**Fig. 3:**
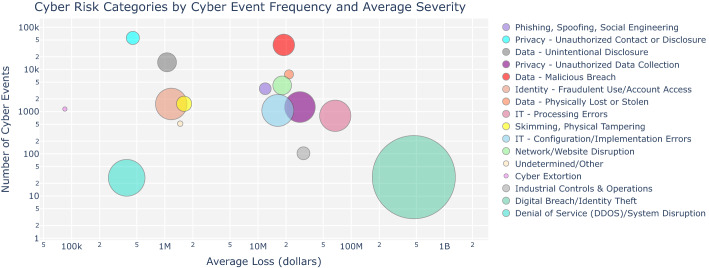
Frequency and severity of individual cyber-related events and the number of affected records (indicated by the size of the circle) across risk categories

Figure 3 shows the Advisen cyber risk threat types ranked by frequency and average severity. Each circle represents a risk category, and the area of the circle corresponds to the average number of records affected. The cyber risk type with the highest average loss and average number of records affected is “Digital Breach/Identity Theft”. Looking at Fig. 3, cyber risk types can be divided into three groups according to their average loss:

1. Average loss lower than USD 2 million: “Cyber Extortion”, “Denial of Service (DDOS)/System Disruption”, “Privacy - Unauthorized Contact or Disclosure”, “Data-Unintentional Disclosure”, “Identity Fraudulent Use/Account Access”, and “Skimming, Physical Tampering”;
2. Average loss between USD 10 million and USD 100 million: “Phishing, Spoofing, Social Engineering”, “IT-Configuration/Implementation Error”, “Network/Website Disruption”, “Data-Malicious Breach”, “Privacy-Unauthorized Data Collection” and “IT-Processing Error”; and
3. Average loss greater than USD 100 million: “Digital Breach/Identity Theft”.

Overall, there seems to be no clear-cut relationship between the frequency of events, loss severity, and the number of affected records. The relationship depends also on the business sector and type of cyber threat.

### Cyber insurance markets

Several interesting works have arisen that study the emerging market for cyber risk insurance, see discussions in Eling and Schnell (2016), McShane et al. (2021) and the editorial and associated special issue of Boyer (2020) and references therein. Recently, McShane et al. (2021) provided a comprehensive review of the literature on managing cyber risks, focusing in particular on work that is related to risk identification, risk analysis, and risk treatment. In particular they noted that despite the emergence of cyber risk studies as far back as 1960, there is still only a fledgling market for cyber risk insurance. Importantly, they highlight many areas that the industry needs to work on in consideration with researchers in the field under the category of “Gaps in the overall cyber risk management process” and they also discuss the importance of cyber resilience, a theme also discussed from a quantitative perspective recently in Xiang et al. (2021).

As discussed in Eling (2018), Eling and Wirfs (2015) and Peters et al. (2018), currently the market for cyber risk insurance is in a state of flux due to uncertainty; in fact it is reported that in practice many companies are favouring forgoing available policies, due to the perceived high cost and confusion about what they cover. Furthermore, there are still several questions arising as to the efficacy of cyber risk insurance, in the sense that creating a market may not provide sufficient coverage of pooling of risk for insurers underwriting such large and uncertain potential losses from this source of risk to remain solvent. In this regard studies that question the suitability of such types of insurance product start to emerge, see discussions in Biener et al. (2015), Peters et al. (2011) and recently in Malavasi et al. (2022).

In Biener et al. (2015) it is noted that as of 2015 the annual gross premiums for cyber insurance in the U.S. are USD 1.3 billion and growing 10–25% on average per year. Furthermore, in continental Europe they claim that cyber insurance products so far are estimated to generate premiums of around USD 192 million, but this figure is expected to reach USD 1.1 billion in 2018. Clearly, this is still a fledgling market compared to other more mainstream lines of insurance business. For such an important and emerging risk class, which is gaining rapidly increasing attention of the banking and finance sector, one may question why these products are still slowly emerging and slowly gaining popularity.

One challenge in this insurance market is the non-standardisation of nomenclature and contract specification of covered items. For instance, products and coverage tend to change rapidly, and exclusions as well as terms and definitions vary significantly between competitors. There is a reason for this flux, primarily it is currently being driven by the fact that the risks faced by corporations are often unique to their industry or even to the company itself, requiring a great deal of customisation in policy writing. This will, we believe, begin to resolve as more data and studies such as the ones we present here begin to emerge highlighting aspects of cyber risk characteristics, see further detailed discussion on this aspect in McShane et al. (2021).

## Data description and attributes

There is an ongoing exploration on the various ways to classify and taxonomise cyber risk loss events, see discussions in Shevchenko et al. (2023), Rea-Guaman et al. (2017) and Elnagdy et al. (2016). In this study the focus has been on the U.S. cyber risk experience as it is generally the environment where the largest commercial cyber loss data collection effort has been instigated, both in terms of breadth of industry and loss type as well as in terms of duration of collection and reporting. In this regard, the paper will focus on the Advisen Cyber Loss Data. This data set provides a historical view of more than 132,126 cyber events from 2008 to 2020, affecting 49,495 organisations across the world. Advisen is a U.S.-based for-profit organisation which collects and processes cyber reports form reliable and publicly verifiable sources such as news media, governmental and regulatory sources, state data breach notification sites, and third-party vendors. Given that the interest in cyber risk is on the rise, many recent studies on cyber risk have made use of Advisen Cyber Loss Data (Romanosky 2016; Cyentia 2020). The understanding and classification of the array of cyber loss event and risk types is diverse and can differ by sector and industry as well as over time.

More than the 80% of the events recorded affect organisations residing in the U.S. and for each event accident timeline, i.e. first notice date, accident date, loss start date, and loss end date, a detailed explanation is reported. One of the key advantages with respect to other commonly used data sets, such as the Chronology of Data Breaches provided by the Privacy Rights Clearinghouse (PRC), is that the Advisen data set gives direct information of monetary losses linked to cyber risk event, providing an empirical measurement of financial losses that can be then used for modelling purposes.

Following Eling and Loperfido (2017) and Edwards et al. (2016) we remove all the observations that do not give information on the monetary losses, and restrict the analysis to the observation for which complete information on company specific characteristics, such as yearly revenue and number of employees are available, leaving the total number of observations considered in this study to 3792, corresponding to roughly the 2.6% of the total. A detailed analysis of the basic attributes, non-statistical in nature, is provided in the industry white paper (Shevchenko et al. 2021) and an overview of how events are classified according to Advisen’s own classification, based on the type of cyber threat, is provided in a detailed overview in Malavasi et al. (2022, Sect. 3).

Other possible classifications of cyber risk events are available in the literature; see Eling and Wirfs (2019) who suggest to divide cyber risk events into categories, according to operational risk classification: Actions by People, System and Technical Failure, Failed Internal Process, External Events. On the other hand, Romanosky (2016) provides cyber risk driven categories, such as Data Breach, Security Incident, Privacy Violation, Phishing Skimming, and Other.

In this work we will instead work with industry-related partitions based on the U.S. NAIC Sector decompositions widely used in insurance practice. The sector-level organisation of NAICs produces 24 unique sector/subsector combinations in which to partition the loss data according to a wide variety of industry types. This classification is one that is widely used in the U.S. industry and provided by the Advisen data providers; in personal correspondence with Advisen’s chief data officer it was advised that industry widely utilises this partitioning of the data and so we opted to study the data from a perspective that would also benefit industry practitioners. Such a cyber risk analysis has not previously been undertaken and we believe this will shed some interesting insight into how different sectors are coping with cyber threats a digital environment.

### Basic empirical data description and attributes

In this section we first provide a basic summary of the Advisen data, first by risk type and then secondly by NAIC sectors. The NAIC sector codes are provided at https://www.naics.com/search/ and at the level 1 categorisation those used by Advisen correspond to: 11 ‘Agriculture, Forestry, Fishing and Hunting’; 21 ‘Mining, Quarrying, and Oil and Gas Extraction’; 22 ‘Utilities’; 23 ‘Construction’; 31 ‘Manufacturing Part A’; 32 ‘Manufacturing Part B’; 33 ‘Manufacturing Part C’; 42 ‘Wholesale Trade’; 44 ‘Retail Trade Part A’; 45 ‘Retail Trade Part B’; 48 ‘Transportation and Warehousing Part A’; 49 ‘Transportation and Warehousing Part B’; 51 ‘Information’; 52 ‘Finance and Insurance’; 53 ‘Real Estate and Rental and Leasing’; 54 ‘Professional, Scientific, and Technical Services’; 55 ‘Management of Companies and Enterprises’; 56 ‘Administration/Support/Waste Management/Remediation Services’; 61 ‘Educational Services’; 62 ‘Health Care and Social Assistance’; 71 ‘Arts, Entertainment, and Recreation’; 72 ‘Accommodation and Food Services’; 81 ‘Other Services (except Public Administration)’; and 92 ‘Public Administration’.

To ensure that records are related to the recent history of cyber risk data, we have excluded from analysis Advisen data that goes back to the 1950s as we are not confident on its accuracy or on its ability to reflect realistic cyber threat environments faced by modern corporations. In this regard we have selected a time window in which we take the earliest reported accident date as 01/01/1990 00:00 through to the most recent accident date of 20/09/2020 00:00. Furthermore, we focus on the analysis of loss records that satisfy that the total loss amount was positive, ignoring many records that register empty or zero cells due to incompletion of the claim or non-settlement or payout. Below in Tables 2 and 3 we show the summary statistics of the data used under each decomposition.

**Table 2:** NAIC sectors and descriptions used by Advisen data to partition loss data into industrial sectors that allow for the study of sector specificity in cyber event data

NAIC Sector	N	Min	Max	Q1	Median	Q3	Mean	St. Dev.	Skew	Kurt
11	2	10,000	2.70M	NA	NA	NA	1.36M	NA	NA	NA
21	7	16,600	7.00M	39,185	299,600	814,000	1.29M	2.55M	1.54	0.64
22	45	30	10.03M	3000	42,337	2.40M	1.51M	2.63M	1.90	2.85
23	62	300	22.41M	6162	32,919	718,663	1.37M	3.90M	3.83	14.81
31	31	6817	84.00M	62,199	310,000	1.80M	4.48M	15.10M	4.70	21.83
32	43	500	410.00M	25,488	231,768	4.20M	20.10M	78.00M	4.21	16.61
33	117	200	2.00B	29,000	487,000	3.30M	27.61M	190.76M	9.57	94.95
42	126	44	2.0B	20,000	237,500	1.8M	0.18M	178M	10.94	118.86
44	280	14	298.00M	10,850	265,887	2.29M	6.98M	31.45M	7.19	54.48
45	126	60	291M	5987	196,250	1.68M	6.57M	28.95M	8.09	73.11
48	51	202	177.00M	55,000	300,000	4.73M	13.19M	37.91M	3.38	10.32
49	12	1632	400.00M	8642	29,101	193,981	33.76M	115.34M	2.65	5.48
51	668	1	5.00B	5290	37,740	1.00M	12.05M	195M	25.05	636.62
52	1027	10	460M	11,608	98,005	1.39M	43.44M	20.95M	13.67	249.46
53	66	1295	28.40M	15,250	132,249	1.20M	132,249	4.33M	4.12	20.20
54	442	89	4.00B	6305	50,180	840,500	17.01M	199.04M	18.45	361.45
55	18	4057	24.69M	15,416	28,736	313,070	2.20M	5.90M	3.06	8.74
56	737	69	1.35B	4500	23,500	1.04M	6.36M	59.50M	17.59	363.81
61	146	800	26.00M	21,250	135,050	726,153	1.03M	2.99M	6.08	42.74
62	289	100	190.00M	26,469	200,000	875,000	2.48M	13.42M	11.51	145.43
71	40	600	30.95M	51,980	933,750	3.80M	4.26M	7.72M	2.10	3.36
72	126	27	226.00M	20,000	160,948	2.28M	6.36M	24.16M	7.03	56.03
81	86	48	90.00M	9183	93,975	688,023	2.88M	11.49M	5.93	38.43
92	384	200	1.0B	20,850	139,300	633,600	5.35M	57.16M	15.41	250.38

The columns represent: N—number of loss events, Mean—average total loss event, Median—typical total loss event, St. Dev.—dispersion of the total loss events, Skew and Kurt.—excess skewness and kurtosis relative to a Gaussian for total loss events

It will also be insightful to see equivalent summary statistics for the total losses also partitioned according to the Advisen risk type classifications, as shown in Table 3.

**Table 3:** This table reports some descriptive statistics of cyber risk related losses aggregated by category, expressed in USD million

Risk Type	N	Mean	Median	St. Dev.	Skew	Kurt
Privacy—Unauthorised Contact or Disclosure	2237	3.698	0.033	25.844	25.197	799.476
Privacy—Unauthorised Data Collection	157	40.283	0.84	401.093	12.171	150.848
Data—Physically Lost or Stolen	93	24.974	0.212	207.490	9.424	90.201
Identity—Fraudulent Use/Account Access	914	1.035	0.028	6.146	10.562	131.617
Data—Malicious Breach	768	20.975	0.5	176.715	17.591	361.688
Phishing, Spoofing, Social Engineering	161	9.219	0.516	59.273	10.726	124.611
IT—Configuration/Implementation Errors	44	6.065	0.804	22.852	5.890	37.472
Data—Unintentional Disclosure	131	2.612	0.250	11.601	9.147	94.133
Cyber Extortion	105	0.634	0.010	3.177	5.998	39.287
Network/Website Disruption	207	7.933	0.090	46.196	7.731	63.533
Skimming, Physical Tampering	87	1.471	0.051	5.930	6.855	54.070
IT—Processing Errors	33	48.872	0.925	120.700	2.826	9.733
Industrial Controls & Operations	5	2.247	0.040	4.359	1.457	3.187
Undetermined/Other	17	1.890	1.500	2.647	3.014	11.627

The losses exhibit great variability in terms of median and first four moments across the risk types. IT—Configuration/Implementation Error, Privacy—Unauthorised Data Collection, and Industrial Controls have the highest average loss amongst all the cyber risk categories

Having explored the basic empirical statistics to summarise the NAIC sector data, we will also now provide some empirical statistical analysis of the data based around three interesting statistical quantities: the smoothed Hill plots of Resnick and Stărică (1997), power law Pareto−based Quantile-Quantile plots, and an extremogram time series analysis of Davis and Mikosch (2009). It is worth noting that previous studies (Maillart and Sornette (2010) and Wheatley et al. (2016)) explored cyber risk modelling using extreme value theory and Pareto type models. However, the focus is on the Catalogue of the Open Security Foundation which provides a representative sample of the overall activity of ID thefts occurring on the Internet and especially for the U.S. for the most important events in terms of the number of ID thefts. This is not a study however on the actual loss amounts, rather it studies the losses of records. This is an important distinction, see the empirical results on this from the Advisen data set in the “Significance of cyber risk losses and cyber insurance markets” section where it is shown empirically in Fig. 2 that monetary losses and the number of records affected vary across business sectors. Business sectors in the top right corner of the graph in Fig. 2 share some common features: they exhibit high average loss and high number of events, and a high average number of records affected (the bubbles have larger sizes than the sector in the top left corner of the graph). This seems to indicate that depending on the intrinsic nature of the business sector, for some sectors there is a connection between a high number of records stolen which translates into high losses. However, for other sectors, a larger number of records does not necessarily translate into greater losses. In our work we focus on the study of the actual loss amounts and the heavy-tailed models associated. Furthermore, we also focus on challenges with the data and how perspectives on trimming and robust estimation of such Pareto tail index estimates can influence perspectives on the insurability of cyber risk losses.

For an ordered independent and identically distributed (i.i.d.) sequence of *n* losses, we will define the increasing sequence of order statistics by $0 < X_{(1,n)}\le X_{(2,n)}\le \cdots \le X_{(n,n)}$ and the decreasing sequence of order statistics by $X^{(1,n)}\ge X^{(2,n)}\ge \cdots \ge X^{(n,n)}>0$, such that $X_{(1,n)} = X^{(n,n)}$ and $X_{(n,n)} = X^{(1,n)}$, the Hill (1975) estimator, discussed in the “Quantifying heavy tails in cyber risk loss models” section, using $k$ order statistics is given by

1 $$\begin{aligned} H_{k,n}=\frac{1}{k}\sum _{i=1}^{k} \ln \left( \frac{X^{(i,n)}}{X^{(k,n)}}\right) , \end{aligned}$$

which is the pseudo-likelihood estimator ($\widehat{\xi }=H^{(k,n)}$) of reciprocal of the tail index $\xi =1/\alpha >0$ for regularly varying tails (e.g. Pareto distribution). Note, when *k* is too small, only a few observations influence $\widehat{\alpha }$ and the variance of the estimator, given asymptotically by $\alpha ^2/k$ is too large. When *k* is too large, the assumption underlying the derivation of the estimator typically degrades and bias increases.

Recall a few basic facts, where a positive measurable function *f* is called regularly varying (at infinity) with index $\alpha \in {\mathbb{R}}$ if it is defined on some neighbourhood $[x_0,\infty )$ of infinity and

$$\begin{aligned} \lim _{x \rightarrow \infty }\frac{f(tx)}{f(x)} = t^{\alpha }, \quad \forall t > 0. \end{aligned}$$

An example of such a distributional law that satisfies the resulting power-law tail behaviour is the Pareto distribution which is given by

$$\begin{aligned} {\mathbb{P}}\left[ X \le x\right] = 1 - \left( \frac{x_m}{x}\right) ^{\alpha } \end{aligned}$$

and which admits a heavy-tailed power law tail in which the tail index $\alpha$ determines the degree of heavy-tailedness. See a detailed discussion on heavy-tailed loss models in Peters and Shevchenko (2015) and the references therein.

The Hill estimator is defined on orders $k>2$, as when $k=1$ the $H^{(1,n)}=0$. Once a sufficiently low order statistic is reached the Hill estimator will be constant, up to sample uncertainty, for regularly varying tails. The Hill plot is a plot of $H^{(k,n)}$ against $k$. Symmetric asymptotic normal confidence intervals assuming Pareto tails are provided. To avoid well known challenges with interpreting the Hill plot, we have opted for the log scale smoothed Hill plot of Resnick and Stărică (1997):

2 $$\begin{aligned} \widetilde{H}_{k,n}=\frac{1}{(r-1)k}\sum _{j=k+1}^{rk} H_{j,n}, \end{aligned}$$

where *r* is the smoothing factor and the order is also on a log scale which is equivalent to plotting the points $(\theta , H^{(\lceil n^\theta \rceil , n)})$ for $0\le \theta \le 1$.

**Fig. 4:**
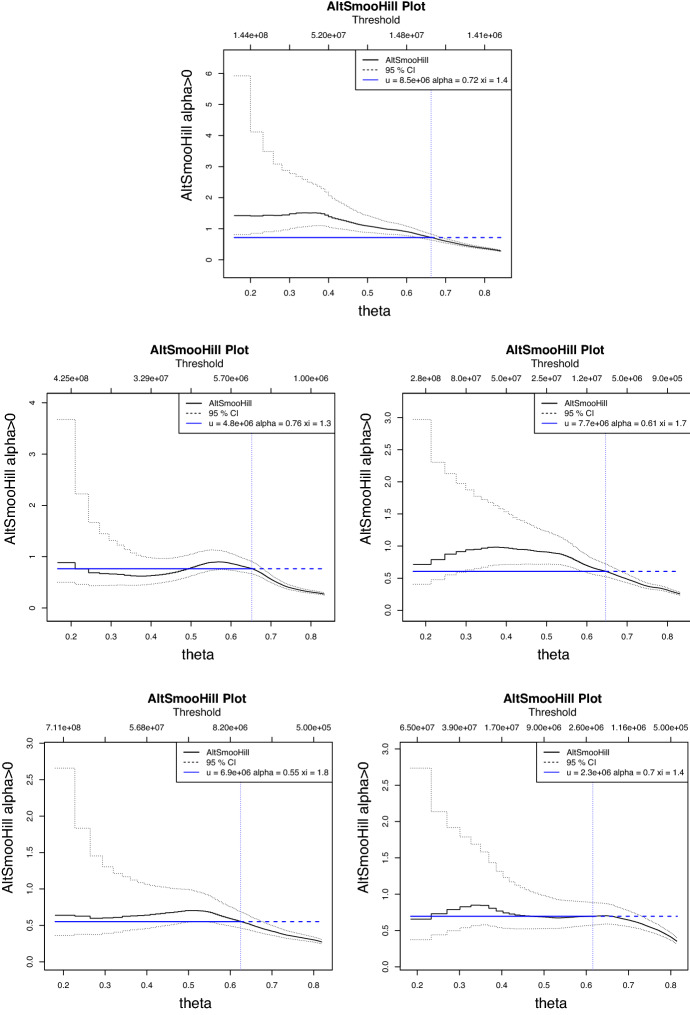
Smoothed Hill plots which show the log scale version of Eq. 2, plotting the points $(\theta , H^{(\lceil n^\theta \rceil , n)})$ for $0\le \theta \le 1.$ Top subplot: NAIC Sector 52. Middle Left subplot: NAIC Sector 56. Middle Right subplot: NAIC Sector 51. Bottom left subplot: NAIC Sector 54. Bottom right subplot: NAIC Sector 92

In Fig. 4 we present the results for the NAIC sectors with the top five largest loss count records corresponding, in order from highest to lowest, to: NAIC = 52 ‘Finance and Insurance’; NAIC = 56 ‘Administrative and Support, and Waste Management and Remediation Services’; NAIC = 51 ‘Information’; NAIC = 54 ‘Professional, Scientific and Technical Services’; and NAIC = 92 ‘Public Administration’. We focus in the studies subsequently undertaken on the top five largest loss count records as, apart from providing sufficient data for model estimation and analysis to be meaningfully applied, they also provided a case study rich enough to illustrate the intended context of the studies undertaken with regard to model risk. Other NAIC category data analysis can be obtained from the corresponding author upon request, but is omitted as a result of space considerations.

**Fig. 5:**
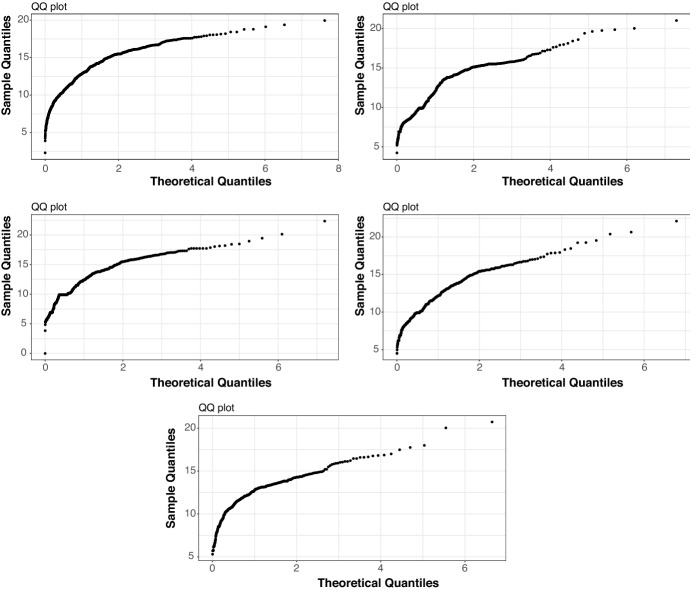
Pareto quantile–quantile plots. Top left subplot: NAIC Sector 51. Top right subplot: NAIC Sector 54. Bottom subplot: NAIC Sector 92

The tail estimation methods proceeding this section will be based on statistical assumptions that relate to heavy-tailed estimation of tail index based on a power law, a regular variation assumption or explicitly a Pareto law or asymptotic Pareto tail behaviour assumptions. Therefore, we also analyse the cyber loss data based on NAIC sectors for Pareto law type behaviour. We will do this in two ways, via Pareto Quantile–Quantile plots as shown in Fig. 5 and following analysis via a hypothesis test.

To conclude this empirical analysis of the leading NAIC categories by total loss amounts, we will explore the extremal correlations as captured by the extremogram. This empirical estimator allows us to capture intertemporal characteristics of the cyber risk loss data categorised by NAIC sector and aggregated over quarterly periods from 1990 to 2021. The extremogram can be considered as a correlogram for extreme events and was introduced originally in works (Davis and Mikosch 2009) as a tool to measure the extremal dependence in ${\mathbb{R}}^d$-valued time series $(X_t)$. The extremogram is defined as a limiting sequence given by

3 $$\begin{aligned} \gamma _{AB}(h)=\lim _{n \rightarrow \infty }\,\text{cov}(I_{\{a_n^{-1} X_0\in A\}} ,I_{\{a_n^{-1} X_h\in B\}}),\quad h\ge 0, \end{aligned}$$

with sequences $(a_n)$ suitably chosen as normalisation sequences and *A*, *B* are two fixed sets bounded away from zero. A popular choice for intervals *A* and *B* is to set $A=B=[q_{\alpha },\infty )$ with $q_{\alpha }$ being the $\alpha$-percentile of $(X_t)$. An example of selection for *A*, *B* that is familiar to the actuarial audience will be to select $A=B=(1,\infty )$ which will reproduce the so-called upper tail dependence coefficient of the vector $(X_0,X_h)$ given as the limit

4 $$\begin{aligned} \rho (h)=\lim _{x \rightarrow \infty } {\mathbb{P}}(X_h>x\mid X_0>x)\,. \end{aligned}$$

In the context used in this work to study cyber risk we will plot a sequence of extremograms marginally for each NAIC sector. Since this method requires regular time series and not an event−driven time record set, we have aggregated the losses into quarterly time series of total losses between 1990 to 2020. For each NAIC sector we produce results for univariate extremograms. We will focus on indexed choices of $A=B \in \left\{ q_{0.01},\ldots ,q_{0.99}\right\}$ corresponding to quantile levels $\alpha \in \left\{ 0.01,\ldots ,0.99 \right\}$. Under this construction, as $\alpha \uparrow 1$ the events $\{X_0\in a_n A\}$ and $\{X_h\in a_n B\}$ are increasingly considered as extreme ones and $\gamma _{AB}(h)$ measures the influence of the time zero extremal event $\{X_0\in a_n\,A\}$ on the extremal event $\{X_h\in a_n\,B\}$, *h* lags apart, i.e. *h* quarters later. This will result in construction of a matrix of values denoted by $\Gamma$, whose *i*, *j*th element is given by $\Gamma _{ij}:=\gamma _{A_i=B_i=q_{\alpha _i}}(j)$ for *j*th quarter from 1990 such that $j \in \left\{ 1,2,\ldots ,124\right\}$ and for quantile thresholds $q_i \in \left\{ 0.01,0.02,\ldots ,0.99\right\}$.

**Fig. 6:**
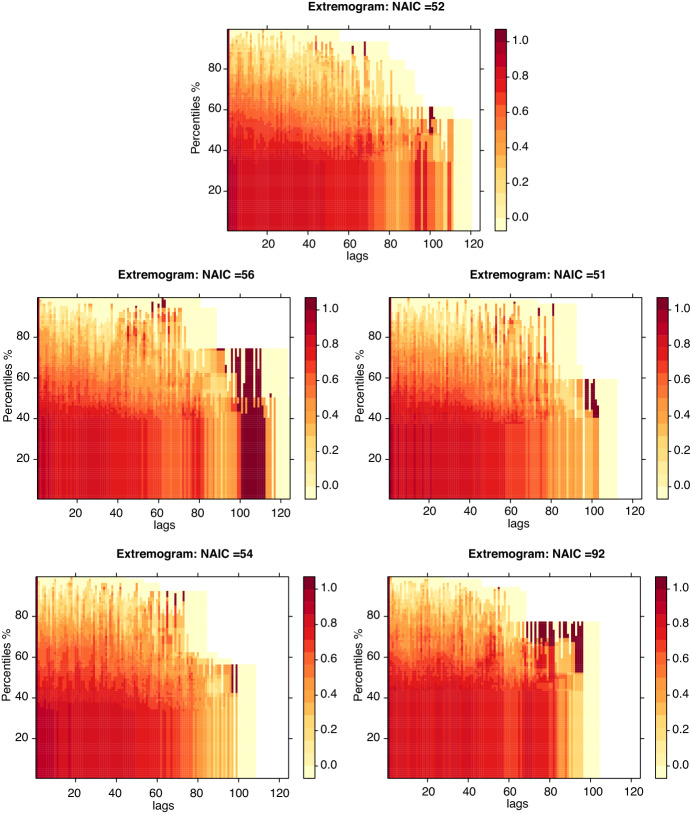
Extremogram plots that show Eq. 3 where points of $(h,\gamma _{AB}(h))$ are plotted for the choices $A=B \in \left\{ q_{0.01},\ldots ,q_{0.99}\right\}$ corresponding to quantile levels $\alpha \in \left\{ 0.01,\ldots ,0.99 \right\}$. Top left subplot: NAIC Sector 52. Top middle subplot: NAIC Sector 56. Top right subplot: NAIC Sector 51. Bottom left subplot: NAIC Sector 54. Bottom right subplot: NAIC Sector 92

We note that in presenting these extremograms in matrix $\Gamma$, whilst the finite quantile sequences for each row *i* always exist for finite $\alpha _i$, the limit as $\alpha _i \uparrow 1$ need not exist. As studied in Davis and Mikosch (2009), it is sufficient for existence of the limits $\gamma _{AB}(h)$ to assume a regularly varying sequence of quarterly loss random variables $(X_t)$, which has power law tails for every lagged vector $(X_1,\ldots ,X_h)$, $h\ge 1$. This assumption, whilst not required to study finite sample extremogram profiles, is required if one wanted to look for extremal asymptotic tail dependence within NAIC sector losses using this methodology. The results of the extremogram analysis for the current selection of NAIC sectors, selected according to the top five largest loss counts, is provided in Fig. 6

## Quantifying heavy tails in cyber risk loss models

In this work we seek to study loss processes which admit heavy-tailed annual loss distribution profiles in the context of cyber risk losses. We are interested in classifying cyber risk losses annually by risk type and business sector. It will be valuable to first explain some basic background on how we will seek to quantify heavy-tailed loss data, both non-parametrically and parametrically with a severity model. We will assume throughout that losses will take a positive support and as such the right tail of the loss distribution is of interest when quantifying heavy-tailed loss behaviour.

There is no unique way to characterise universally the notion of a heavy-tailed distribution, and as such numerous definitions and characterisations have been proposed. We will explore a few key characterisations in this section, starting with a widely used concept that a heavy-tailed loss model is characterised via the existence of moments. Under this characterisation, a heavy-tailed loss model *F* will not have finite moments of some order, and the heavier the tail, the fewer moments will exist. A simple condition that shows this relationship is stated in the following Lemma 4.1.

### Lemma 4.1

The distribution *F* possesses an absolute moment of order $\alpha > 0$ if and only if (iff) $|x|^{\alpha -1}\left[ 1-F(x) + F(-x)\right]$ is integrable over $(0,\infty )$.

Another way to characterise heavy-tailed models that is also often explored in risk modelling theory, is to state that heavy-tailed distributions are probability distributions whose tails are not exponentially bounded: that is, they have heavier tails than the exponential distribution. Under this characterisation, one considers those distributions for which the moment-generating function does not exist on the positive real line such that

$$\begin{aligned} \int e^{sx}dF(x) = \infty , \; \forall s > 0. \end{aligned}$$

In other words, take the standard Markov’s inequality with $\psi$ a monotonically increasing non-negative function for the non-negative reals, *X* is a random variable, $a \ge 0$, and $\psi (a) > 0$, then applying

$$\begin{aligned} {\mathbb{P}}(|X|\ge a)\le \frac{{\mathbb{E}}(\psi (|X|))}{\psi (a)} \end{aligned}$$

for the exponentially decaying ‘light’ tail behaviour of a loss distribution

$$\begin{aligned} \overline{F}(x) \le \exp (-sx){\mathbb{E}}\left[ \exp (sX)\right] , \quad \forall x > 0 \end{aligned}$$

does not apply.

In addition to multiple characterisations, there are also numerous ways to represent and study a heavy-tailed loss distribution, beyond just the distribution function, that will be useful to briefly recall.

### Definition 4.1

(Hazard Function and Hazard Rate) For a loss distribution *F* on ${\mathbb{R}}^+$, the hazard function is given by

$$\begin{aligned} R(x) = -\ln \overline{F}(x). \end{aligned}$$

If the loss distribution *F* has a loss density *f*, then such a distribution’s tail behaviour can be characterised also by the hazard rate, given by

$$\begin{aligned} r(x) := \frac{dR(x)}{dx} = \frac{f(x)}{1-F(x)} = \frac{f(x)}{\overline{F}(x)} = -\frac{\overline{F}_X(x)'}{\overline{F}_X(x)}. \end{aligned}$$

### Lemma 4.2

(Hazard Rate of a Loss Distribution) Hazard Rate following three right limiting possibilities of the hazard rate function of a loss distribution to characterise its tail behaviour:

1. If $\lim _{x \rightarrow \infty } r(x) = 0$, then the loss distribution *F* will be a heavy-tailed distribution function.
2. If $\lim _{x \rightarrow \infty } r(x) > 0$, then the loss distribution *F* is not heavy-tailed and the exponential moments will exist up to $\lim \inf _{x \rightarrow \infty } r(x)> \lambda > 0$
3. If $\lim _{x\rightarrow \infty } r(x)$ does not exist but one has $\lim \inf _{x \rightarrow \infty } r(x) = 0$, then the distribution *F* can be either heavy or light tailed, and one needs further information to determine the characteristics.

One can summarise the relationship between different representations of a heavy-tailed loss distribution as follows [Foss et al. (2011), Theorem 2.6]:

### Theorem 1

For any distribution *F*, the following assertions are equivalent:

- *F* *is a heavy-tailed distribution.*
- $\overline{F}$ *survival function is heavy tailed.*
- *Corresponding hazard function* *R* *satisfies* $lim \, inf _{x \rightarrow \infty } R(x)/x = 0$.
- *For some fixed *$T > 0$, *the function F on interval* $(x,x+T]$ *is heavy tailed.*
- *If distribution* *F* *is absolutely continuous with density function* *f* *then if F* *is heavy tailed, the density* *f* *is also heavy tailed.*

There are numerous more refined categorisations of heavy-tailed distributions, we will recall an important class of heavy-tailed loss models, those that correspond to the regularly varying tail behaviour characterisation.

### Definition 4.2

(Regularly Varying Tail) A probability distribution *F* has regularly varying tails, $\overline{F} \in {\mathcal {R}}$ iff for some $\alpha \ge 0$ and any $y > 0$, it holds that

$$\begin{aligned} \lim _{x\rightarrow \infty }\frac{\overline{F}(xy)}{\overline{F}(x)} = y^{-\alpha }. \end{aligned}$$

We say a function *L*(*x*) is slowly varying if $L \in {\mathcal {R}}$ and $\alpha = 0$.

If we consider the characteristic function of loss random variable *X*, or of *F*, is $\varphi$, defined for all *t* by

$$\begin{aligned} \varphi (t) = \int _{-\infty }^{\infty } \exp \left( itx\right) dF(x), \end{aligned}$$

then another way to characterise the heavy-tailed nature of the loss random variable is through the relationship between the value of *F*(*x*) for large *x* and the value of $\varphi (t)$ for small *t* in the neighborhood of the origin. We will briefly rewrite the characteristic function as follows:

$$\begin{aligned} \varphi (t)=\int _{-\infty }^{\infty }\cos (tx)dF(x) + i \int _{-\infty }^{\infty }\sin (tx)dF(x) = U(t) + i V(t). \end{aligned}$$

Now, we will consider the distribution function re-expressed in terms of the tail sum $H(x) = 1 - F(x) + F(-x)$ which in the case of loss distribution models in which the support is positive reduces to $H(x)=\overline{F}(x)$.

To proceed, we will assume $\overline{F}(x)$ is regularly varying at infinity, i.e.

$$\begin{aligned} \overline{F}(x) = x^{-\alpha }L(x), \quad \text {as } x \rightarrow \infty , \end{aligned}$$

where $\alpha > 0$ and *L*(*x*) is slowly varying at infinity. Now consider the characteristic function of *X* for all real *t* split into real and imaginary components and integrate by parts to obtain

$$\begin{aligned} 1 - U(t) = t\int _{0}^{\infty }\sin (tx)\overline{F}(x)dx. \end{aligned}$$

which means that the behaviour of the tail sum depends only on *U*(*t*), the real part of the characteristic function. To see this proceed as follows:

$$\begin{aligned} \varphi (t)&= \int _{-\infty }^0 e^{itx}dF(x) + \int _{0}^{\infty } e^{itx} d\left[ F(x)-1\right] \\&= \int _{-\infty }^0 \cos (tx)dF(x) + \int _{0}^{\infty }\cos (tx)d[F(x)-1] \\&\quad + i\left\{ \int _{-\infty }^0 \sin (tx)dF(x) + \int _{0}^{\infty }\sin (tx)d[F(x)-1]\right\} . \end{aligned}$$

Integrating by parts gives

$$\begin{aligned} \varphi (t)&= F(0) + t \int _{-\infty }^0 \sin (tx)F(x)dx \\& \quad - (F(0)-1) + t\int _{0}^{\infty }[F(x)-1]\sin (tx)dx \\& \quad - it \left\{ \int _{-\infty }^0 \cos (tx)F(x)dx + \int _{0}^{\infty }\cos (tx)[F(x)-1]dx\right\}  \end{aligned}$$

which then allows one to obtain for the real component of the characteristic function $\varphi (t)$ the identity

$$\begin{aligned} U(t) - 1&= t \int _{-\infty }^0 \sin (tx)F(x)dx + t \int _{0}^{\infty } [F(x)-1]\sin (tx)dx\\&= -t \int _{0}^{\infty } \sin (tx)F(-x)dx + t\int _{0}^{\infty } [F(x)-1]\sin (tx)dx\\&= -t \int _{0}^{\infty } \sin (tx)H(x)dx. \end{aligned}$$

This result was a critical part of the work of Pitman (1968) who went on to show that for infinite variance loss random variables with tail sum function *H*(*x*), of index of regular variation $0<\alpha <2$, that as $x \rightarrow \infty$, one has the following relationship between the real part of the characteristic function near the origin and the regularly varying tail function

$$\begin{aligned} 1 - U(t) \sim s(\alpha )H(1/t) = s(\alpha )L(1/t)t^{\alpha }, \quad \text { as } t\downarrow 0, \end{aligned}$$

with

$$\begin{aligned} s(u) = {\left\{ \begin{array}{ll} \frac{\pi /2}{\Gamma (u)\sin (u\pi /2)}, &{} \quad \text {if}\; u >0 \\ 1, &{} \quad \text {if} \; u=0, \end{array}\right. } \end{aligned}$$

with *s*(*u*) finite for any *u* not an even positive integer. Other cases for $\alpha =2$ and $\alpha > 2$ were studied but are not directly of relevance to the work in this paper where we concentrate to heavy-tailed models with non-finite variance or non-finite mean.

Hence in summary, as $t \downarrow 0$, depending on value of tail index parameter $\alpha$ one can obtain the relationship

5 $$\ln \left( 1 - U(t)\right) = \left\{ \begin{array}{ll} \ln \left( s(\alpha ) L(1/t)\right) + \alpha \ln t, & \quad \text {if}\; 0<\alpha <2\\ \ln \left(\int\limits_0^{1/t}xH(x)dx\right) + 2 \ln t, & \quad \text{if}\; \alpha =2\\ \ln \left( \frac{\mu _2}{2}\right), & \quad \text{if}\; \alpha >2. \end{array}\right.$$

This identity is the precursor to tail estimators such as Hill estimators, which we will use to study in the non-parametric analysis of cyber risk loss data in the “Quantifying heavy tails in cyber risk loss models” section. To understand this, consider taking loss data and estimating the real part of the empirical characteristic function using the observed cyber risk loss samples $\left\{ X_1, \ldots , X_n \right\}$ assumed i.i.d. from *F* to produce

$$\begin{aligned} \varphi _n(t) = \frac{1}{n} \sum _{j=1}^n \exp \left( i t X_j\right) \end{aligned}$$

and therefore the empirical estimator for the real component is $U_n(t) = \frac{1}{n} \sum _{j=1}^n \cos \left( t X_j\right)$. From this one can then define the empirical quantity for a grid of values $t_1, \ldots , t_N$ around the origin which produce tuples $\left\{ \left( t_i,\ln \left[ 1-U_n(t_i)\right] \right) \right\}$ which can be regressed given a general assumption about the slowly varying tail function *L* to produce an estimator for the tail index $\widehat{\alpha }_n$ based on linear regression obtained in the general form

6 $$\begin{aligned} \ln \left( 1-U_n(t_j)\right) \sim \ln C + \alpha \ln t_j + \ln \frac{1- U_n(t_j)}{1- U(t_j)} , \quad j = 1, 2,\ldots , m, \end{aligned}$$

where one can assume that this looks like a simple linear regression with $y_i = \ln \left( 1-U_n(t_j)\right)$, $Z_j = \ln t_j$ and error $\epsilon _j = \ln \frac{1- U_n(t_j)}{1- U(t_j)}$ which then admits a least squares estimator for $\alpha$. This method is interesting as it is basically non-parametric and relies upon an estimator formed from the empirical characteristic function around the origin. Note that in such estimators for the tail index $\alpha$, the bias in the estimation will be directly related to the assumption regarding the model for *L*(*x*) which clearly enters through *U*.

Other methods of similar nature make more specific assumption about *H*(*x*) and *L*(*x*) or at least about the asymptotic functional form of these quantities as $x \rightarrow \infty$. This naturally then leads to classes of estimators such as the Hill estimator (Hill 1975) and generalisations such as those discussed in an excellent work exploring aspects of tail index estimation by Hall (1982). This basic observation of using an asymptotic relationship and a simple linear regression to estimate the tail index can be studied in a plethora of other related approaches, we will not review all of these in this work. Our focus in this work will be on two classes of general estimator for tail index consistent with a regular variation assumption, those based on characteristic function asymptotics and those based on maximum likelihood principle. Within these two categories there are in fact more than 100 currently known estimators for the tail index based various different assumptions about *H*(*x*) and its tail behaviour, see a comprehensive account of many of these estimators in Fedotenkov (2020), a very comprehensive and well written review of univariate Pareto-type tail index estimators for i.i.d. non-truncated data. With regard to the maximum likelihood based approaches, the classic approach most widely used by practitioners is known as the Hill estimator. We discuss in this paper a few different variants of this estimator that can treat bias in the cyber risk data collection and robustness considerations.

In both contexts we will consider application of extensions that build upon the work of Hall (1982) where it was proposed to consider particular types of tail sum generic functional forms of the slowly varying function *L*(*x*) that will parametrise $\overline{F}(x) = x^{-\alpha }L(x)$ as $x \rightarrow \infty$ in the regularly varying tail function class,

7 $$\begin{aligned} L(x) = C\left[ 1 + Dx^{-\beta } + o(x^{-\beta })\right] \end{aligned}$$

for $C>0$, $\alpha > 0$, $\beta > 0$ and *D* is a non-zero real number, which gives tail sum function

8 $$\begin{aligned} H(x) = Cx^{-\alpha }\left[ 1 + Dx^{-\beta } + o(x^{-\beta })\right] \;\text{as}\; x\rightarrow \infty . \end{aligned}$$

Note this type of parametric form captures:

- Stable distributions with stability index $\alpha \in (1,2)$ by setting $\beta /2 < \alpha \le \beta$,
- Extreme value distributions with $F(x) = \exp \left( -x^{-\alpha }\right)$ for $x > 0$ and $\alpha = \beta$,
- Powers of “smooth” distributions of loss *X* where if $X = Y^{-1/\alpha }$ then *Y*’s distribution admits a Taylor series expansion of at least three terms about the origin.

In the following sections we will explore in detail working with the choice of regularly varying model assumptions consistent with the power-law type severity models such as stable Pareto-Levy or Pareto type heavy-tailed loss models. To achieve this we will work with estimators of the tail index $\alpha$ for loss data based on empirical regression based estimators in one of two forms, either based on empirical characteristic function regressions near the origin or on assumptions on the likelihood used to derive MLE based estimators like Hill estimators. Furthermore, we will also consider some robust versions of the Hill estimator recently developed that extend the classical Hill type estimators aforementioned to accommodate removal of potentially biased or misreported massive losses, subject to significant uncertainty, rounding error, non-actual payment, reporting error and more.

### Tail index estimators for cyber risk based on empirical characteristic function asymptotic regressions

If one makes the assumption of Hall in Eq. 7 then it was shown by Pitman (1968) and Welsh (1986) that if Eq. 8 is assumed then as $x \rightarrow \infty$ one can express the resulting asymptotic real component of the power-law distributions characteristic function as follows near the origin as $t \downarrow 0$:

9 $$\begin{aligned} 1 - U(t) = C s(\alpha ) t^{\alpha } + D_1t^{\gamma } + o(t^{\gamma }), \end{aligned}$$

where $\alpha \in (0,2)$ and it satisfies for a non-negative integer *p* the constraint $2p< \alpha + \beta < 2p + 2$, with $\gamma = \min \left\{ \alpha + \beta , 2\right\}$ and constant $D_1 = CD s(\alpha + \beta )$ if $\alpha + \beta < 2$, see discussion in Jia (2014) for more general characterisations that extend the representations for arbitrary real $\alpha$.

If one then substitutes this representation based on Eq. 7 for $\alpha \in [0,2]$ then as $t \downarrow 0$ one obtains a resulting refinement to the asymptotic relationship in Eq. 6, derived in Jia (2014, Sect. 2.2) given for $y = \ln (1-U(t))$ by:

10 $$\begin{aligned} y&= C_{\alpha } + \alpha \ln t + \ln \frac{1-U_n(t)}{1-U(t)}, \\ C_{\alpha }&= \ln \left[ C s(\alpha )\right] + \frac{D_1}{C s(\alpha )}t^{\gamma -\alpha } + o(t^{\gamma -\alpha }), \end{aligned}$$

where $C_{\alpha }$ is treated as the constant of the regression and the estimator of the tail index given by simple least squares produces:

11 $$\begin{aligned} \widehat{\alpha } & = \frac{\sum _{j=1}^m a_j y_j}{S_{zz}},\\ \widehat{C}_{\alpha } &= \overline{y} - \widehat{\alpha } Z, \end{aligned}$$

with $a_j = Z_j - \overline{Z} = \ln t_j - \frac{1}{m}\sum _{k=1}^m \ln t_k$ and $S_{zz} = \sum _{i=1}^m \left( Z_i - \overline{Z}\right) ^2 = \sum _{i=1}^m a_i^2$. For further properties of this estimator including its mean squared error, guidance on optimal selection of points for the regression around the origin see details in Jia (2014, Sect. 2.3). We will use these properties in forming the analysis of the Advisen data and we therefore provide briefly a few key properties for practitioners below.

#### Theorem 2

Suppose that *H*(*x*) the tail sum for the loss distribution characterizing the cyber loss severity distribution satisfies the assumptions of Hall (Hall 1982), such that

12 $$\begin{aligned} H(x) = Cx^{-\alpha }\left[ 1 + Dx^{-\beta } + o(x^{-\beta })\right] \text { as } x\rightarrow \infty . \end{aligned}$$

For the heavy-tailed case of $\alpha \in (0,2)$ and estimators given by Eq. 11, with $t_j = j/\sqrt{n}$ for $j=1,\ldots ,m = n^{\delta }$ with $\delta \in (0,1/2)$. Then as $n \rightarrow \infty$ the bias of this regression based tail index estimator is given by

13 $$\begin{aligned} {\mathbb{E}}\left[ \widehat{\alpha }\right] - \alpha = \frac{D_1(\gamma -\alpha )}{Cs(\alpha )(\gamma -\alpha +1)^2}n^{(\delta -1/2)(\gamma -\alpha )}\left\{ 1 + o(1) \right\} , \end{aligned}$$

and the variance of the estimator is given by

14 $$\begin{aligned} {\text{Var}}\left( \widehat{\alpha }\right) = O\left( n^{\alpha /2 - 1 - \delta \alpha }\right)  \end{aligned}$$

where the exponent power for the variance is always less than 0.

The proof for these results is provided in Jia (2014).

### Tail index estimators for cyber risk based on Hill-type estimators

A second popular approach to tail index estimation that we will explore for cyber risk data, applicable when one is willing to assume an additive tail function *H*(*x*) which is Pareto in law, is the class of Hill estimators. There are many variations on the Hill estimator; we will first show the basic form of the estimator and then discuss briefly important variations that make the estimator more statistically robust. Numerous authors have contributed to authors have contributed to the development of this class of estimators, see for instance influential works by Hill (1975), Pickands (1975), Hall (1982), Embrechts et al. (2013) and the references therein.

Suppose that $X_1,\ldots ,X_n$ is an i.i.d. sample from a heavy-tailed distribution *F*. Namely,

15 $$\begin{aligned} {\mathbb{P}}(X_1> x) \equiv 1-F(x) \sim \ell (x) x^{-1/\xi }\quad \text{ as } x\rightarrow \infty , \end{aligned}$$

for some $\xi >0$ and a slowly varying function $\ell :(0,\infty )\rightarrow (0,\infty )$, i.e. $\ell (\lambda x)/\ell (x)\rightarrow 1,\ x\rightarrow \infty ,$ for all $\lambda >0$. The parameter $\xi$ is also often referred to as the *tail index* of *F* and it is typically treated in this context as equivalent to $\alpha$ in previous sections being represented as $\alpha = 1/\xi$. It will be convenient in this cyber risk study to use both tail index notations: with $\alpha$ to refer to the regression type estimator based on the characteristic function, derived above; and $\xi$ to refer to the class of Hill estimators based on maximum likelihood estimation under an asymptotic Pareto power law assumption for the observed losses. At this point it will be informative to also recall the classic *Hill estimator* given by the below, which is the estimator of tail index $\xi$, which is the inverse of the tail exponent $\alpha$:

16 $$\begin{aligned} \widehat{\xi }_{k,n}:=\widehat{H}_{k,n}=\frac{1}{k} \sum _{i=1}^{k} \ln \left(\frac{x^{(i,n)}}{x^{(k,n)}} \right)= \widehat{\alpha }^{-1}. \end{aligned}$$

Fortunately, Munasinghe et al. (2022) has produced a useful R package that implements a range of tail index estimators of Hill “type” which we will loosely refer to as the variety of related estimators based on the asymptotic Pareto power law assumptions for cyber risk losses. In all estimation methods based on extremal order statistics, one must determine a threshold to begin using the order statistics in the estimator since the tail index working under the assumption of Pareto distributed data either exactly or asymptotically. Therefore, to apply these methods for the general power law form, we would look to identify where tail behaviour starts, which is not a precise or easy task, the interested reader is referred to Hill (1975), Hubert et al. (2013), Vandewalle et al. (2007) for further detail and Fedotenkov (2020) for a catalogue of Pareto-tail index estimation techniques.

The different Hill type estimators we will consider to use will be:

1. *Maximum Likelihood Estimation (MLE)* the MLE formula gives an estimator for inverse $\hat{\xi }$:
  17 $$\begin{aligned} \hat{\alpha } = N \left[ \sum _{i=1}^n \frac{x_i}{\hat{x}_{\text{min}}} \right] ^{-1}, \end{aligned}$$
  where $x_i$ represents the data point for $i=1, \ldots , n$. The minimum value, $x_{\text{min}} = x_{(1,n)}$, is estimated from the data set and hence denoted $\hat{x}_{\text{min}}$. As noted in Newman (2005) this leads to a biased estimator, however this estimate (17) can be converted to an unbiased version $\alpha ^{\ast}$ as follows (Rizzo 2009):
  18 $$\begin{aligned} \alpha ^{\ast} = \frac{n-2}{n} \hat{\alpha }. \end{aligned}$$
2. *Weighted variants of Least Squares Estimation (WLS)* This method is based on the order statistics, assumed sorted in increasing order. Then for each value *i* (of *n* data points) one calculates $y_i$ the number of points greater than the *i*th data point. This method seeks to minimise the sum of the squared errors between the rank plot and the logarithm of the cdf. The estimator is given by Nair (2013):
  19 $$\begin{aligned} \hat{\alpha } = \frac{ \sum _{i=1}^n \left( \hat{y_i} - \frac{1 }{n} \sum _{i=1}^n \hat{y_i} \right) \left( \ln x_i - \frac{1}{n} \sum _{i=1}^n \ln x_i \right) }{ \sum _{i=1}^n \left( \ln x_i - \frac{1}{n} \sum _{i=1}^n \ln x_i \right) ^2}. \end{aligned}$$
  There is also a popular weighted variant where the sum of squared errors criterion from the LS method above is developed with a weight function. A common choice of weight function is given by
  20 $$\begin{aligned} w_i = \left[ \ln \left( \frac{x_i}{\hat{x}_{\text{min}}} \right) \right] ^{-1} \end{aligned}$$
  which gives a WLS solution closely related to the first estimator based on the MLE where each has the same asymptotic limiting results. Under this weight function the WLS tail index estimate is then given, assuming no ties in the sorted losses, by
  21 $$\begin{aligned} \hat{\alpha } = -\frac{ \sum _{i=1}^n \ln \left[ (n+1-i) / n \right] }{ \sum _{i=1}^n \ln \left( x_i / \hat{x}_{\text{min}} \right) }. \end{aligned}$$
3. *Percentile Method (PM)*: this method develops an estimator for the tail index based on percentiles, typically based on a robust dispersion measure such as the inter-quartile range, producing estimators such as (see Bhatti et al. 2018):
  22 $$\begin{aligned} \hat{\alpha } = \frac{ \ln 3}{ \ln (P^{\ast}_{75}) - \ln (P^{\ast}_{25})}. \end{aligned}$$
  Here $P^{\ast}_{q}$ is the *q*th percentile of the data set.

**Fig. 7:**
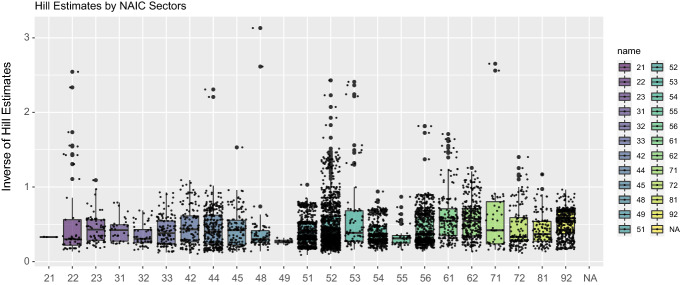
Box plots of Hill estimators obtained from a sequence of thresholds corresponding to percentile levels of the data from N–k to N

We begin the results analysis by looking at the basic Hill estimator obtained for a sequence of order statistic thresholds *k*. The results are presented in Fig. 7 and Table 4.

**Table 4:** Tail index estimators for each NAIC sector. N.A. means that the sample size was insufficient for a reliable estimator to be obtained

Sector NAIC	Emp.Char.Fn Reg.	MLE	LS	MoM	PM	MPM	GPM	WLS
11	N.A.	(0.3572,10000)	(0.0724,10000)	N.A.	(0.1962,10000)	(1.7252,10000)	(0.1538,10000)	(0.1238,10000)
21	N.A.	(0.3840,16600)	(0.2228,16600)	N.A.	(0.3004,16600)	(0.5329.16600)	(0.2430,16600)	(0.2796,16600)
22	N.A.	(0.1332,30)	(0.1930,30)	N.A.	(0.1586,30)	(0.1692,30)	(0.1006,30)	(0.1216,30)
23	N.A.	(0.1879,300)	(0.2663,300)	N.A.	(0.2210,300)	(0.2158,300)	(0.1494,300)	(0.1761,300)
31,32,33	0.0612 ± 0.1574	(0.1353,200)	(0.2638,200)	N.A.	(0.2292,200)	(0.2860,200)	(0.1623,200)	(0.1319,200)
42	0.0013 ± 0.0263	(0.1228,44.49)	(0.2423,44.49)	N.A.	(0.2433,44.49)	(0.3396,44.49)	(0.1557,44.49)	(0.1188,44.49)
44,45	0.0083 ± 0.0192	(0.1235,40.58)	(0.2320,40.58)	N.A.	(0.2028,40.58)	(0.3295,40.58)	(0.1417,40.58)	(0.1217,40.58)
48, 49	N.A.	(0.1295,202)	(0.2411,202)	N.A.	(0.2380,202)	(0.2454,202)	(0.1604,202)	(0.1217,202)
51	0.0457 ± 0.0326	(0.0900,1)	(0.2551,1)	N.A.	(0.2081,1)	(0.2115,1)	(0.1444,1)	(0.0876,1)
52	0.0077 ± 0.0128	(0.1068,10)	(0.2783,10)	N.A.	(0.2288,10)	(0.2607,10)	(0.1550,10)	(0.1060,10)
53	N.A.	(0.2140,1295)	(0.2869,1295)	N.A.	(0.2464,1295)	(0.3080,1295)	(0.1752,1295)	(0.2040,1295)
54	0.0059 ± 0.0141	(0.1478,88.98)	(0.2770,88.98)	N.A.	(0.2238,88.98)	(0.2453,88.98)	(0.1614,88.98)	(0.1457,88.98)
55	N.A.	(0.3237,4056.84)	(0.2355,4056.84)	N.A.	(0.2565,4056.84)	(0.1976,4056.84)	(0.1625,4056.84)	(0.2811,4056.84)
56	0.0243 ± 0.0183	(0.1475,69.04)	(0.2826,69.04)	N.A.	(0.2017,69.04)	(0.1827,69.04)	(0.1358,69.04)	(0.1457,69.04)
61	0.0197 ± 0.0210	(0.1986,800)	(0.3470,800)	N.A.	(0.3040,800)	(0.4067,800)	(0.2147,800)	(0.1925,800)
62	N.A.	(0.1341,100)	(0.3368,100)	N.A.	(0.3098,100)	(0.4634,100)	(0.2360,100)	(0.1312,100)
71	N.A.	(0.1508,600)	(0.2335,600)	N.A.	(0.2395,600)	(0.4771,600)	(0.1778,600)	(0.1391,600)
72	0.0078 ± 0.0275	(0.1126,27)	(0.2391,27)	N.A.	(0.2289,27)	(0.2554,27)	(0.1534,27)	(0.1092,27)
81	N.A.	(0.1367,47.95)	(0.2384,47.95)	N.A.	(0.2461,47.95)	(0.3386,47.95)	(0.1672,47.95)	(0.1310,47.95)
92	0.0029 ± 0.0162	(0.1586,200)	(0.3158,200)	N.A.	(0.3193,200)	(0.4543,200)	(0.2185,200)	(0.1564,200)

The following index estimators $\alpha$ are presented:

Emp.Char.Fn.Reg: Empirical Characteristic Function regression with point estimator $\alpha$ ± std. error;

MLE: Maximum Likelihood Estimators of Hill for tail index and scale parameters such that $(\alpha , scale)$;

LS: Least Squares Estimator with no weighting for tail index and scale parameters such that $(\alpha , scale)$;

MoM: Method of Moments—not relevant as all instances were too heavy-tailed to admit reliable MoM estimators;

PM: Percentile Method estimator for tail index and scale parameters such that $(\alpha , scale)$;

MPM: Modified Percentile Method estimator for tail index and scale parameters such that $(\alpha , scale)$;

GPM: Generalised Percentile Method estimator for tail index and scale parameters such that $(\alpha , scale)$;

WLS: Weighted Least Square estimator for tail index and scale parameters such that $(\alpha , scale)$

We note that for the empirical characteristic function regression estimator, we utilised the results in Theorem 2 to select the values for $t_1,\ldots ,t_m$ such that $t_j = j/\sqrt{n}$ for $m = n^{\delta }$ with $\delta = 1/2$ which was adaptive for each NAIC sector as they have differing numbers of realised cyber losses. It is evident from this analysis firstly that there is significant variation in the estimators across the various methods of tail index estimation. The Method of Moments (MoM) failed in all cases due to the sample size requirements and the Empirical Characteristic Function Regression methods were also seemingly unable to produce reliable results in numerous NAIC examples and in those in which it did produce estimator results, the uncertainty associated with the estimators under this method in this cyber risk application were very large. The MLE, PM, MPM, GPM and WLS were seemingly better at capturing estimators that were more comparable with each other across each NAIC dataset. These estimators indicated the presence of very heavy-tailed loss distributions for the majority of NAICs. This type of finding is consistent with other studies of tail behaviour in cyber risk loss data. In the following section, we will question and explore the validity of these findings, which if taken at face value, would indicate a difficulty with insurability due to the heavy-tailed nature of the loss processes that would result in exorbitant premiums. We will therefore explore if the situation is as bad as it looks by taking a more practical perspective on the analysis and by exploring assumptions regarding the loss data recording and the impact they may have on such conclusions regarding heavy-tailed behaviour of cyber loss processes.

## Dealing with real world cyber data: inaccurate, rounded, truncated, partially settled unreliable massive reported cyber total losses

In real cyber risk loss data, the total loss may be subject to a range of issues in the reporting. The attribution of all loss components to the total loss may be difficult when it concerns a combination of both direct and indirect loss aggregations. General reporting of cyber risk, in the financial sector for instance, falls under two broad classifications of loss type: direct and indirect losses.

- Direct losses: Resulting from the event itself, such as reparation, time lost, client compensation, regulatory fines, money lost in wrongful transactions.
- Indirect losses: Resulting from the consequences of the event such as loss of customers resulting from damage to image or reputation, low morale amongst employees, regulatory scrutiny, increased insurance premiums. Indirect losses are often linked to reputation damage!

We adopted the Advisen data set categorisations of each loss event into direct and indirect losses. In the Advisen data studied in this paper the total cyber loss per event is actually a composition of many direct and indirect loss components including: injury loss payouts awarded, loss of wages, loss of business income, loss of assets, property first-party payouts, financial damages, loss of life expense payouts, defence costs for legal and regulatory, other expenses, punitive exemplary damages, other fines and penalties, pain and suffering awarded amounts, other costs, plaintiff legal fees and plaintiff fees. It is observed across the different cyber risk types that the proportion of direct and indirect losses is highly heterogeneous, see an overview available from the Advisen data in Table 5 and further discussion and analysis in Shevchenko et al. (2023).

**Table 5:** Summary of losses in USD by risk category in Advisen data

Risk type		Loss amounts decomposed according to indirect and direct losses categories (according to Advisen in USD for U.S.)										
		Loss of wages	Loss business income	Financial damages	Defense costs	Other expenses	Punitive exemplary damages	Multiplied damages	Fines penalties	Pain and suffering	Other	Plaintiff legal fees
Privacy Unauthorized Contact or Disclosure	Min	409,350	7568	100	400	200	500	1000	200	250	700,000	175
	25% Quantile	518,402	861,284	4500	3100	20,100	4500	1000	4000	5205	1,025,000	4347
	Median	798,388	1,715,000	73,000	77,000	40,000	29,000	1000	20,000	42,000	1,350,000	99,500
	Mean	2,028,024	167,240,856	6,087,000	3,038,600	33,915	1,809,006	1000	1,514,628	573,062	1,350,000	898,517
	75% Quantile	1,712,875	250,857,500	2,200,000	3,112,500	50,773	142,250	1000	275,000	210,664	1,675,000	756,474
	Max	9,000,000	500,000,000	2,673,000,000	12,000,000	61,454	51,999,960	1000	112,500,000	3,537,450	2,000,000	21,815,550
Privacy Unauthorized Data Collection	Min	NA	16,430	1	NA	884,000	400,000	NA	47	100,000	NA	55,000
	25% Quantile	NA	961,606	22,500	NA	884,000	400,000	NA	109,200	100,000	NA	160,246
	Median	NA	2,161,695	600,000	NA	884,000	400,000	NA	275,000	100,000	NA	356,000
	Mean	NA	2,084,955	10,343,751	NA	884,000	400,000	NA	123,600,000	533,333	NA	994,081
	75% Quantile	NA	3,285,044	2,691,700	NA	884,000	400,000	NA	2,050,000	750,000	NA	1,117,411
	Max	NA	4,000,000	650,000,000	NA	884,000	400,000	NA	5,000,000,000	1,400,000	NA	9,103,088
Data Physically Lost or Stolen	Min	NA	231,768	380	NA	NA	NA	NA	3000	NA	3000	25,000
	25% Quantile	NA	231,768	55,000	NA	NA	NA	NA	100,000	NA	35,804	90,799
	Median	NA	231,768	101,500	NA	NA	NA	NA	325,000	NA	1,000,000	225,000
	Mean	NA	231,768	43,390,000	NA	NA	NA	NA	850,310	NA	5,836,387	519,526
	75% Quantile	NA	231,768	700,000	NA	NA	NA	NA	926,803	NA	3,175,000	712,500
	Max	NA	231,768	2,000,000,000	NA	NA	NA	NA	4,348,000	NA	53,480,000	2,312,066
Identity Fraudulent Use /Account Access	Min	NA	234,000	10	NA	NA	70,000	NA	2,100	NA	44,938	114,250
	25% Quantile	NA	250,500	4,000	NA	NA	115,000	NA	206,707	NA	46,179	209,515
	Median	NA	267,000	24,500	NA	NA	160,000	NA	250,000	NA	47,420	300,000
	Mean	NA	267,000	13,560,000	NA	NA	160,000	NA	17,262,399	NA	47,420	1,388,753
	75% Quantile	NA	283,500	181,000	NA	NA	205,000	NA	4,563,760	NA	48,661	2,820,000
	Max	NA	300,000	11,400,000,000	NA	NA	250,000	NA	127,500,000	NA	49,902	3,500,000
Data Malicious Breach	Min	NA	10,000	25	NA	NA	100,000	NA	1,100	NA	450	90
	25% Quantile	NA	67,129	60,000	NA	NA	150,000	NA	129,708	NA	50,000	192,500
	Median	NA	225,000	407,000	NA	NA	200,000	NA	636,720	NA	500,000	552,500
	Mean	NA	19,828,858	23,840,000	NA	NA	4,433,333	NA	4,759,029	NA	21,390,000	2,443,764
	75% Quantile	NA	987,324	2,700,000	NA	NA	6,600,000	NA	1,892,486	NA	5,600,000	1,587,500
	Max	NA	304,000,000	4,000,000,000	NA	NA	13,000,000	NA	425,000,000	NA	1,000,000,000	40,950,000
Phishing, Spoofing, Social Engineering	Min	NA	192,883	800	NA	NA	NA	NA	25,000	NA	10,000	3,500
	25% Quantile	NA	192,883	156,734	NA	NA	NA	NA	452,500	NA	58,230	95,662
	Median	NA	192,883	539,111	NA	NA	NA	NA	1,275,000	NA	127,000	150,000
	Mean	NA	192,883	4,421,008	NA	NA	NA	NA	90,496,644	NA	4,311,504	966,996
	75% Quantile	NA	192,883	1,000,000	NA	NA	NA	NA	3,962,500	NA	303,500	957,500
	Max	NA	192,883	225,277,500	NA	NA	NA	NA	710,738,150	NA	51,000,000	4,509,150
IT Configuration/ Implementation Errors	Min	NA	209,443	1,258	NA	NA	NA	NA	100,000	NA	50,357	8,508
	25% Quantile	NA	1,969,582	234,833	NA	NA	NA	NA	197,873	NA	90,228	106,833
	Median	NA	3,729,722	562,600	NA	NA	NA	NA	308,908	NA	130,100	106,835
	Mean	NA	3,729,722	7,419,474	NA	NA	NA	NA	868,726	NA	4,393,486	723,962
	75% Quantile	NA	5,489,861	1,860,000	NA	NA	NA	NA	1,775,000	NA	6,565,050	262,400
	Max	NA	7,250,000	150,000,000	NA	NA	NA	NA	2,140,500	NA	13,000,000	7,450,000
Data Unintentional Disclosure	Min	NA	NA	170	NA	220,000	NA	NA	250	NA	1,200	510
	25% Quantile	NA	NA	10,000	NA	220,000	NA	NA	15,000	NA	30,000	42,666
	Median	NA	NA	168,739	NA	220,000	NA	NA	59,058	NA	88,320	138,750
	Mean	NA	NA	1,402,553	NA	220,000	NA	NA	941,121	NA	389,916	1,081,763
	75% Quantile	NA	NA	938,750	NA	220,000	NA	NA	575,000	NA	580,750	481,250
	Max	NA	NA	21,630,000	NA	220,000	NA	NA	23,900,000	NA	1,600,000	26,851,000
Cyber Extortion	Min	NA	9,800,000	30	NA	NA	NA	NA	NA	NA	1,500,000	NA
	25% Quantile	NA	9,800,000	2,050	NA	NA	NA	NA	NA	NA	1,500,000	NA
	Median	NA	9,800,000	25,000	NA	NA	NA	NA	NA	NA	1,500,000	NA
	Mean	NA	9,800,000	944,032	NA	NA	NA	NA	NA	NA	1,500,000	NA
	75% Quantile	NA	9,800,000	116,000	NA	NA	NA	NA	NA	NA	1,500,000	NA
	Max	NA	9,800,000	40,000,000	NA	NA	NA	NA	NA	NA	1,500,000	NA
Network/ Website Disruption	Min	NA	54,006	234	NA	NA	NA	NA	65,438	NA	1000	325,000
	25% Quantile	NA	124,293	10,000	NA	NA	NA	NA	491,500	NA	42,250	325,000
	Median	NA	2,100,000	50,000	NA	NA	NA	NA	925,000	NA	295,000	325,000
	Mean	NA	51,297,234	7,969,851	NA	NA	NA	NA	2,929,680	NA	8,516,885	370,753
	75% Quantile	NA	18,650,000	344,005	NA	NA	NA	NA	1,900,000	NA	2,025,000	370,753
	Max	NA	410,000,000	652,900,000	NA	NA	NA	NA	16,000,000	NA	320,000,000	508,011
Skimming, Physical Tampering	Min	NA	NA	316	NA	600,000	NA	NA	50,000	NA	14,324	95,000
	25% Quantile	NA	NA	11,500	NA	600,000	NA	NA	50,000	NA	5,088,243	923,750
	Median	NA	NA	48,000	NA	600,000	NA	NA	62,500	NA	10,162,162	1,600,000
	Mean	NA	NA	1,077,830	NA	600,000	NA	NA	231,250	NA	10,162,162	4,573,750
	75% Quantile	NA	NA	304,500	NA	600,000	NA	NA	243,750	NA	15,236,081	5,250,000
	Max	NA	NA	35,000,000	NA	600,000	NA	NA	750,000	NA	20,310,000	15,000,000
IT Processing Errors	Min	NA	33,750	26,500	NA	NA	NA	NA	15,000	NA	300	60,000
	25% Quantile	NA	44,275,312	71,875	NA	NA	NA	NA	62,500	NA	667,006	105,000
	Median	NA	88,516,875	8,100,000	NA	NA	NA	NA	270,000	NA	5,744,011	150,000
	Mean	NA	88,516,875	28,723,062	NA	NA	NA	NA	918,055	NA	84,424,720	378,333
	75% Quantile	NA	132,758,438	28,625,000	NA	NA	NA	NA	545,300	NA	17,250,000	537,500
	Max	NA	177,000,000	265,000,000	NA	NA	NA	NA	7,000,000	NA	475,000,000	925,000
Industrial Controls & Operations	Min	NA	NA	40,000	NA	NA	NA	NA	33,000	NA	30,000	NA
	25% Quantile	NA	NA	313,707	NA	NA	NA	NA	2,524,750	NA	30,000	NA
	Median	NA	NA	587,414	NA	NA	NA	NA	5,016,500	NA	30,000	NA
	Mean	NA	NA	587,414	NA	NA	NA	NA	5,016,500	NA	30,000	NA
	75% Quantile	NA	NA	861,121	NA	NA	NA	NA	7,508,250	NA	30,000	NA
	Max	NA	NA	1,134,828	NA	NA	NA	NA	10,000,000	NA	30,000	NA
Undetermined/ Other	Min	NA	NA	36,000	NA	NA	NA	NA	30,000	NA	NA	NA
	25% Quantile	NA	NA	52,000	NA	NA	NA	NA	122,664	NA	NA	NA
	Median	NA	NA	68,000	NA	NA	NA	NA	575,000	NA	NA	NA
	Mean	NA	NA	68,000	NA	NA	NA	NA	1,474,700	NA	NA	NA
	75% Quantile	NA	NA	84,000	NA	NA	NA	NA	1,937,500	NA	NA	NA
	Max	NA	NA	100,000	NA	NA	NA	NA	11,510,000	NA	NA	NA
Digital Breach/ Identity Theft	Min	NA	NA	200,000	NA	NA	NA	NA	NA	NA	NA	NA
	25% Quantile	NA	NA	15,050,000	NA	NA	NA	NA	NA	NA	NA	NA
	Median	NA	NA	110,000,000	NA	NA	NA	NA	NA	NA	NA	NA
	Mean	NA	NA	267,550,000	NA	NA	NA	NA	NA	NA	NA	NA
	75% Quantile	NA	NA	362,500,000	NA	NA	NA	NA	NA	NA	NA	NA
	Max	NA	NA	850,000,000	NA	NA	NA	NA	NA	NA	NA	NA

Indirect and direct loss categories for which data was available. Note: NA is data unavailable from Advisen

Through correspondence with Advisen on data veracity and providence, they as the data provider acknowledged that all these losses are difficult to accurately measure, record and obtain information on over multiple events that make up a total loss event for a given accident trigger. As such we conclude that this can make it likely that when losses of one billion. or more are recorded for total loss, they are often less specific and rounded compared to those losses observed in the thousands and millions. In addition, it is also the case that not all total losses are settled—they may be awarded but in practice if they are in the billions they will likely not ever be completely settled. This causes uncertainty in the extreme losses used to estimate the Hill estimator, which are the most critical losses for accurate tail index estimation. In this section we will explore how to overcome this challenge. In practice, if the largest few order statistics are *corrupted* or *unreliable* or *uncertain* as just discussed, this may lead to severe bias in the estimation of $\xi$. In the worst case scenarios one may find that the computed estimate of $\xi$ may be completely constructed from a small number of corrupted observations. To reduce the effect of such observations that may corrupt the sample one may introduce a class of Hill estimators based on trimming and weighting that produces a class of robust tail index estimators.

### Robust trimmed Hill estimators for cyber losses

To reduce this bias several authors have looked at how to robustify the Hill estimator, see Brazauskas and Serfling (2000), Zou et al. (2020), Goegebeur et al. (2014), Peng and Welsh (2001) and Peng and Welsh (2001). The objective of these methods was to robustify the estimator of $\xi$ by trimming or reducing the reliance on the potentially corrupted extreme losses; this can be done through a hard truncation, weighting or a soft truncation weighted trimming method. We note that since such methods are often data driven, when selecting the degree of trimming, rather than utilising a cyber specific input, such methods outlined below are also suitable for other areas of severe loss insurance modelling such as in natural catastrophe modelling.

The trimmed Hill estimator, denoted ${\mathcal {H}}_{k_0,k,n}$ below, is based on a weighted version of the classical Hill estimator, for some selection of weights $\left\{ w_{k_0,k}(i)\right\}$ for the order statistics between $i \in \left\{ k_0,\ldots ,n\right\}$. The selection of the weighting rule will clearly influence the statistical properties of the estimator obtained, generically given by:

23 $$\begin{aligned} \widehat{\xi }^{\text{trim}}_{k_0,k,n}:= {\mathcal {H}}_{k_0,k,n} = \sum _{i=k_0+1}^{k} w_{k_0,k}(i) \ln \left(\frac{X_{(n-i+1,n)}}{X_{(n-k,n)}} \right), 0\le k_0<k<n-1. \end{aligned}$$

It is worthy to remark that when $k_0<k<n$ then one has set the weight contributions from the potentially corrupted higher order statistic losses through to zero contribution after adjusting for the intermediate order statistics from $k_0$ onwards, which are incorporated in the estimation with a weighting rule. The consequence of this approach is to improve the breakdown point of the robust estimator. However, the selection of $k_0$ is a delicate matter and cannot be readily determined apriori. Therefore it is proposed to consider utilising a trimmed Hill plot to help select the choice of the trimming parameter $k_0$ which is of key importance in practice. One can utilise a method of the *trimmed Hill plot* to visually determine $k_0$. Then, by exploiting the elegant joint distribution structure of the optimal trimmed Hill estimators, Bhattacharya et al. (2017) devised a weighted sequential testing method for the identification of $k_0$. This leads to a new *adaptive trimmed Hill* estimator, which works well even if the degree of contamination in the top order statistics is largely unknown.

Inference for the *truncated* Pareto model has been developed in the seminal work of Aban and Meerschaert (2004) and recently in Beirlant et al. (2016). In contrast to this, Bhattacharya et al. (2017) studied a soft truncation approach based on weighted trimming in which the class of weights that would be optimal in the sense of being the Best Unbiased Linear Estimator (BLUE) for the class of loss models given by a regularly varying Pareto law and were able to obtain a closed form representation for the weighting functions.

It was demonstrated in Bhattacharya et al. (2017) that in the ideal Pareto setting, it turns out that our trimmed Hill estimator is essentially finite–sample optimal among the class of all unbiased estimators of $\xi$ with a fixed *strong upper breakdown point*. Furthermore, they established the following properties of the estimator for $\xi$:

- Asymptotic normality of the trimmed Hill estimator in the semiparametric regime (15), under second order conditions on the regularly varying function $\ell$ as in Beirlant et al. (2004).
- Rate of convergence of the estimator being the same as the classic Hill as long as $k_0=o(k)$.

The optimal BLUE trimmed weights, $w_{k_0,k}(i)$ for which the estimator in (23) is unbiased for $\xi$ and also has the minimum variance produces the tail index estimator given by

24 $$\begin{aligned} \widehat{\xi }^{\text{trim\, opt}}_{k_0,k,n} &\,{:=}\, {\mathcal {H}}_{k_0,k,n}=\frac{k_0+1}{k-k_0} \ln \left(\frac{X_{(n-k_0,n)}}{X_{(n-k,n)}} \right)\\ & \quad +\frac{1}{k-k_0} \sum _{i=k_0+2}^{k} \ln \left(\frac{X_{(n-i+1,n)}}{X_{(n-k,n)}} \right), \quad 0\le k_0<k<n-1 \end{aligned}$$

see Bhattacharya et al. (2017) for details. Furthermore, Bhattacharya et al. (2017) proposed a data driven parameter selection procedure for the threshold $k_0$ selection.

The potential uncertainty regarding the validity of extreme losses reported not only causes bias in the estimation of the tail parameter $\xi$, it is also reasonable to assume that such uncertainty could translate into premium mispricing. Basing insurance premium calculations on the trimmed Hill estimators should reduce the impact of uncertainty and provide for more robust premium estimates. When the trimmed Hill estimates show some consistency, perhaps suggesting values of the tail parameter greater than 1, one could then conclude that the heavy tails of cyber risk are mainly caused by a few extreme losses that may be inaccurately reported or recorded or suffer from a great degree of uncertainty in their assessment, that could be corrupted or even never settled, and therefore there should be little to no variation in the corresponding insurance premiums when trimming these noisy records. On the other hand, if the values of the trimmed Hill estimates still showed no consistency, then one should conclude that the effect of uncertainty in the cyber-related losses is not mitigated by the trimming procedure, implying presence of further model risk in the case of cyber risk related to inconsistency of the cyber loss process with the statistical assumptions underlying the tail index estimators. To investigate this, we consider a simple insurance premium calculation, including in our analysis the trimmed Hill estimator.

Consider a one-year insurance policy protecting against each of $X_1,\ldots ,X_n$ random losses, with *n* following a Poisson distribution, up to an aggregate top cover limit equivalent to a percentage *c* of the total company wealth. According to the zero utility principle, the maximum premium *P* a non-satiable and risk averse decision maker, with total wealth *w* is willing to pay, corresponds to the solution of the following non linear equation:

25 $$\begin{aligned} {\mathbb{E}}\left[ u\left( w-\sum _{i=1}^n X _i\right) \right] = {\mathbb{E}}\left[ u\left( w-P -\sum _{i=1}^n X_i + \sum _{i=1}^n\min (X_i,cw)\right) \right] , \end{aligned}$$

where $u(x)=\widetilde{u}(\max (x,1))$ and $\widetilde{u}(\cdot )$ is a concave, non-decreasing utility function. We consider a company with USD 1 billion of total wealth, wishing to insure $10\%$ of its total capital. For $k = 1,\ldots ,20$, and $k_0 = 15,\ldots ,60$ with step size of 5, we estimate the tail parameter using the trimmed Hill estimator in 24, and fit quarterly frequency on a Poisson distribution for each NAIC. Then, we proceed with insurance premium calculations using a simulation framework under this illustrative example.

**Fig. 8:**
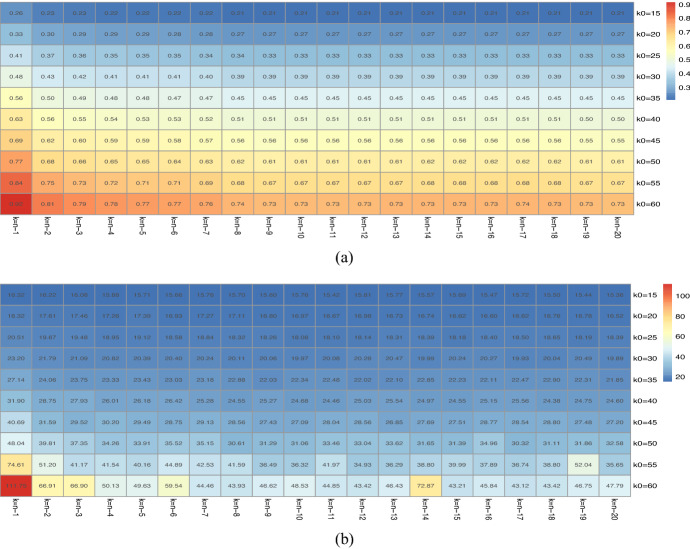
Trimmed tail index estimator $\widehat{\xi }^{\text{trim\, opt}}_{k_0,k,n}$ obtained by trimmed Hill estimators (on the top) and corresponding insurance premiums (on the bottom) for varying trimming parameters $(k_0,k)$ for NAIC 52. Premiums are computed on a quarterly basis using 1000,000 Monte Carlo draws. The variation in the trimmed Hill estimates translates into significant variation in the resulting insurance premium calculations

We present the results for the leading NAIC by total cyber losses in Fig. 8. The remaining 4 NAICs that are in focus have similar results which are presented in Appendix 1, see Fig. 13. These plots show the trimmed Hill estimates for various values of *k* and $k_0$ (a) and the corresponding insurance premiums (b), of the top five NAIC sectors in terms of cyber event frequency.

All the considered NAICs exhibit a variation in the estimates of the trimmed tail index, and we see that critically, this variation indeed then translates into variation in the insurance premiums required. This shows that depending on what model assumptions one is willing to make regarding the quality of the data used in tail index estimation, these assumptions have a consequential influence on insurance pricing. This manifests as a form of model risk in dealing with cyber risk data, given that the effect of uncertainty cannot be filtered out by trimming procedures.

Importantly, we see that business sectors NAIC52 and NAIC56 both show trimmed inverse tail parameter estimates which now appear substantially lower than 1, suggesting that the extreme tail behaviour reported in Fig. 7 and Table 4 is mainly driven by a few extreme losses. According to the assumptions underpinning the application of the trimming methodology, outlined previously, one can see that according to the application of this technique, it yields a switch from a heavy-tailed model to a lighter-tailed loss model. In other words, by changing the assumptions on the cyber risk loss data, one goes from a non-trimmed class of estimates which produces heavy-tailed loss models for insurance pricing through to the trimmed estimates which produced lighter-tailed loss models and the resulting consequences on the insurance premiums is substantial. We see for NAIC52 that this results in a difference in premiums in which the premium reduces by up to 86% under the trimmed model assumptions compared to the non-trimmed. For NAIC56 (results presented in Appendix 1) there is also a substantial premium change as trimming is applied, and in this case it also results in a shift from heavy-tailed loss model to light-tailed loss model that subsequently results in a reduction in premium of up to 93%, a very substantial difference in premium pricing. We note that in the case of these two NAIC examples, we state that the model has shifted from heavy-tailed to light tailed, since the results shift for any initial $k_0$ immediately from a heavy-tailed to a light tailed model as soon as any trimming is applied and the inverse tail index continues to decrease as increasing trimming is applied. Importantly in both cases, it is seen that after a certain point of trimming, for a lower threshold $k_0$, the trimmed estimates stabilise indicating that one can reliably fit a model to this data once problematic, noisy, inaccurate or corrupted data is removed.

The results for NAIC51, NAIC54 and NAIC92 are presented in Appendix 1, and whilst these models still have heavy-tailed loss models after trimming is applied, the resulting premium reductions from applying the trimmed results compared to the non-trimmed results is also substantial. The maximum premium reduction produced for NAIC51 was a 99.3%, for NAIC 54 it was 99.5% and for NAIC92 it was of 99.6%. These are so substantial that they clearly indicate the need to consider this source of model risk and the potential impact on pricing coming from the underlying modelling assumption and subsequent model risk.

Tail index estimates for business sectors NAIC51, NAIC54 and NAIC92 still suggest that the corresponding severity distributions are heavy-tailed. Nevertheless, this is not consistent for every value of *k* and $k_0$, implying that the estimates are highly sensitive to the choice of *k* and $k_0$. The sensitivity of the tail index estimates directly translates into insurance premiums. For business sectors NAIC52 and NAIC56, insurance premiums computed using a log-utility function appear to be lower than those for business sectors NAIC51, NAIC54 and NAIC92. However, all business sectors present great variability in insurance premiums, showing how uncertainty in the tail index ultimately affects premium mispricing and cyber risk insurability.

## Dependence and tail behaviour estimation on Advisen NAIC cyber losses

In this section we will illustrate that in addition to model risk and parameter uncertainty on the marginal loss processes, manifesting in insurance pricing uncertainty, one can also find in the setting of cyber risk data significant model risk and parameter uncertainty in the joint dependence model between cyber loss processes, which again we will study across the NAIC industry sectors.

Analogously to the analysis in the previous section that studied marginal tail behaviours under different data and model assumptions, in this section we will repeat this type of analysis but for the dependence structures between NAIC cyber loss processes. This will be performed in a sequence of stages, starting with a comparison between simple linear correlation estimates and various robust correlation estimators. Then we will develop this further to account for copula models under various assumptions and we will select optimal copula dependence structures. In terms of how these various studies of dependence manifest in an insurance context for cyber risk, we will explore the impact that parameter uncertainty, model misspecification and model risk in the dependence structures may have on risk diversification for insurers that may hold insurance portfolios for cyber risk across many industry sectors as captured by the NAIC codes.

**Fig. 9:**
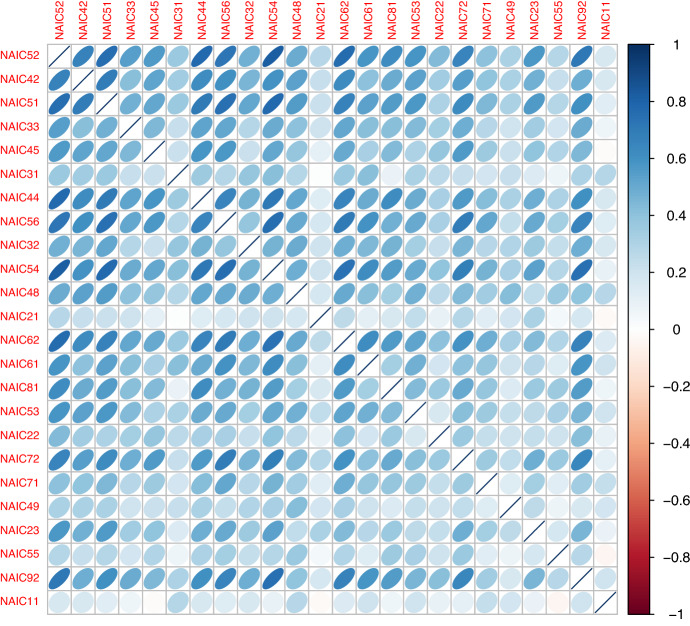
Standard linear Pearson correlation coefficient (Eq. 26) estimates for quarterly aggregate historical Advisen Cyber Loss Data across NAIC sectors in the U.S. between 01/01/1990 and 20/09/2020

We begin this section by comparing standard linear correlation estimates between the loss data for each NAIC in the Advisen data. We will then go on to demonstrate how the correlation estimation may be effected by robust estimators that make different assumptions on the data when calculating the linear or rank correlations. In Fig. 9 we present the basic linear correlations between NAIC industry sectors. Note, throughout this section we will need to convert the loss data from event time series data where losses have time stamps on the days of loss event, to a regular time series in order to compare dependence structures between NAICs. To achieve this we have decided after analysis of the data records that a reasonable time stratification is to perform a quarterly aggregation for the dependence analysis.

The Pearson linear correlation coefficient is given as follows

26 $$\begin{aligned} r = \frac{\sum (x_i - \overline{x})(y_i-\overline{y})}{\left[ \sum (x_i-\overline{x})^2\sum (y_i-\overline{y})^2\right] ^{1/2}}, \end{aligned}$$

which in this analysis uses all loss events in the quarterly aggregates. This includes the indirect and direct loss events that for the extreme loss records were highly likely to suffer from different forms of inaccuracy, ranging from noisy reporting due to approximations, inaccurate records and rounding, misreporting or incomplete reporting, partially settled or unsettled and corrupted records, which is particularly relevant for the extreme loss in the Advisen data, as discussed in the “Dealing with real world cyber data: inaccurate, rounded, truncated, partially settled unreliable massive reported cyber total losses” section.

To determine how such issues may result in model uncertainty or model risk in this analysis, we will once again compare the standard linear Pearson correlation coefficient estimators, ignoring these problems with the extreme loss records to estimators for dependence that are robust and can remove the influence of such problematic large loss records to various degrees, depending on the class of robust estimator. We will explore three robust methods of dependence estimation for correlation based on SSD Median, Quadrant (sign) correlation coefficient methods and MCD estimators, each outlined below. In developing the analysis for the robust correlation estimation, we once again focus on the most important NAICs, studied in previous sections, that have the top five number of loss events reported: NAICs 52, 56, 51, 54, 92.

As discussed in Shevlyakov and Smirnov (2011) one can robustify the sample correlation coefficient by replacing the linear procedures of averaging by the corresponding non-linear robust counterparts according to

27 $$\begin{aligned} r_{\alpha }(\Psi ) = \frac{\sum _{\alpha }\Psi (x_i - \widehat{x})\Psi (y_i - \widehat{y})}{\left[ \sum _{\alpha } \Psi ^2(x_i-\widehat{x})\sum _{\alpha } \Psi ^2(y_i - \widehat{y})\right] ^{1/2}}, \end{aligned}$$

where $\widehat{x}, \widehat{y}$ are robust estimators of location such as the median that are used to replace the mean. This is particularly important in the case of infinite mean loss models. The function $\Psi (\cdot )$ is a monotonic function such as Huber’s $\Psi$-function given by

28 $$\begin{aligned} \Psi (z,k) = \max \{ -k, \min (z,k)\} \end{aligned}$$

and $\sum _{\alpha }$ is a robust version of the data summation that can trim values as follows

29 $$\begin{aligned} \sum _{\alpha }z_i = nT_{\alpha }(z) = n(n-2r)^{-1}\sum _{i=r+1}^{n-r} z_{(i,n)}, \quad 0\le \alpha \le 0.5, \quad r=[\alpha (n-1)], \end{aligned}$$

with $[\cdot ]$ the integer component. Note, when $\alpha = 0$ one recovers the standard summation and no trimming of order statistics is applied. If one wishes to recover the classical correlation median estimators of Falk (1998) one can select $\alpha = 0.5, \widehat{x} = {\text{med}}(x), \widehat{y} = {\text{med}}(y)$ and $\Psi (z)=z$, where ${\text{med}}(z)=z_{([n/2],n)}$ and we will study a version of this with trimming of extremes.

Furthermore, we will utilise a non-parametric measure for robust correlation of Blomqvist (1950) known as the quadrant (sign) correlation coefficient given by $\alpha =0$, $\widehat{x}={\text{med}}(x),\quad \widehat{y}={\text{med}}(y)$ and $\Psi (z) = {\text{sgn}}(z)$ to produce estimator

30 $$\begin{aligned} r_{Q} = n^{-1}\sum {\text{sgn}}(x_i - {\text{med}}(x)){\text{sgn}}(y_i - {\text{med}}(y)). \end{aligned}$$

The final robust correlation estimator we will explore will be the Minimum Covariance Determination (MCD) estimator. This is obtained for a finite sample of observations $\left\{ x_1 , \ldots , x_n\right\}$ in ${\mathbb{R}}^p$ by selecting that subset $\left\{ x_{i_1}, \ldots , x_{i_h}\right\}$ of size *h*, with $1 \le h \le n$, which minimises the generalised variance given by the determinant of the covariance matrix computed from the subset among all possible subsets of size *h*. The resulting robust location and scale estimators are then defined as

31 $$\begin{aligned} \widehat{x}&= \frac{1}{h}\sum _{j=1}^h x_{ij},\\ \widehat{\Sigma }&= c_p \frac{1}{h}\sum _{j=1}^h (x_{ij}-\widehat{x})(x_{ij}-\widehat{x})^T, \end{aligned}$$

where $c_p$ is a consistency factor. The location estimator can also be replaced with a robust M-Estimator such as the median estimator for the trimmed sample. The choice $h = [(n+p+1)/2]$ is commonly preferred since it yields the highest possible breakdown point, see Lopuhaa et al. (1991). As these authors observed, setting it atleast as high as $h \approx n/2$ when the number of observations is much higher than the dimension means the breakdown point of the resulting multivariate scale estimator is defined as the smallest fraction of observations that you need to replace to arbitrary position before the estimated scatter explodes such that its largest eigenvalue tends to infinity or implodes such that its smallest eigenvalue tends to zero.

**Fig. 10:**
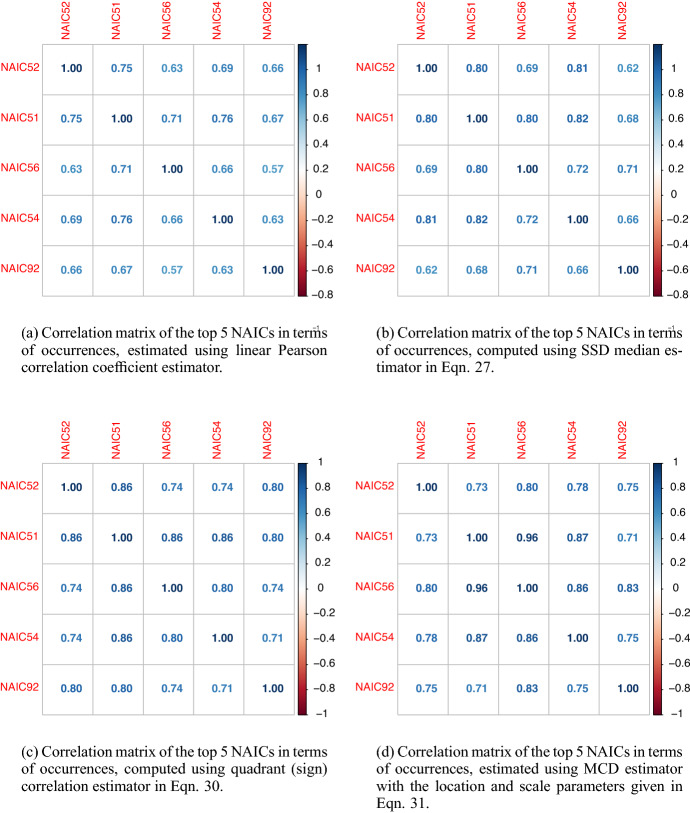
This figure shows the correlation matrices of NAIC52, NAIC51, NAIC56, NAIC54, and NAIC92. Estimates for correlation varies between the different estimators suggesting model risk also affects the dependence structure of cyber event severity

Figure 10 shows the correlation matrices for cyber event severity occurring in the top five NAICs in terms of number of events. Looking at the correlation estimates, the linear correlation case presents lower coefficients than the other robust estimators. This suggests that linear correlation might underestimate the strength of the dependence structure in cyber event severity. Moreover, the observed high degree of variation between the robust correlation estimates might suggest that cyber event severity dependence structure is also affected by parameter uncertainty.

We further investigate the impact of uncertainty in cyber event severity dependence parameters on insurance pricing, using a zero utility principle. In this case study, we consider a hypothetical insurance company with multiple lines of business for their cyber risk insurance policies. Each line of business corresponds to insurance policies issued to companies in the U.S. categorised under a given NAIC industry sector. This insurance company will then have a portfolio of insured cyber risks across various industry sectors. We will be interested in assessing in this section the influence on such an insurance portfolio of the model risk and parameter uncertainty associated with estimation of the dependence structure between the different cyber risk loss processes by line of business or NAIC. To continue the working illustration, we will focus on an insurance portfolio corresponding to the five NAICs studied in previous sections: NAIC52, NAIC 51, NAIC 56, NAIC54 and NAIC92. We will then modify the zero utility equation in (25) as follows to accommodate this insurance portfolio context, accounting for the dependence structures present between the NAICs, as shown in Eq. 32,

32 $$\begin{aligned} {\mathbb{E}}\left[ u\left( w-\sum _{i=1}^5\sum _{n=1}^{N_i}\omega _iX^i _n\right) \right] = {\mathbb{E}}\left[ u\left( w-P -\sum _{i=1}^5\sum _{n=1}^{N_i}\omega _iX_n^i+\sum _{i=1}^5\sum _{n=1}^{N_i}\omega _i\min (X_n^i,cw)\right) \right] , \end{aligned}$$

where $\omega _i$ corresponds to the weight of each NAIC *i*. Note, we use notation $N_i$ here to denote the fact that it is a random variable for the number of losses in a given year. In order to numerically solve Eq. 32 one needs to know the joint distribution of cyber event frequency and severity occurring in the five considered NAICs. While it’s possible to employ copulas to approximate the multivariate compound process, in case of insurance premium calculations this might pose some challenges, given the presence of the top cover limit. Moreover, correlation estimates in Fig. 10 refer to quarterly aggregated losses. A possible solution is to implement an extension to more dimensions of the algorithm in Cruz et al. (2015, Chap. 12), where losses are drawn from a distribution of correlated aggregated losses. We will outline a summary of this approach as follows.

Consider a d-dimensional compound loss process ${\textbf{Z}}=\left[ Z^1,\ldots ,Z^d\right]$, with each component a compound loss given by $Z^i=\sum _{n=1}^{N^i} X_n^i$, where $N^i\sim F_{N^i}$ and $X_n^i\sim F_{X^i}$, $n=1,\ldots ,N^i$ are random variables corresponding to the frequency and severity of the cyber event occurring in NAIC *i*. The joint distribution of ${\textbf{Z}}$, $F_{{\textbf{Z}}}$ can then be uniquely expressed using a copula *C* and the marginal distributions of each component, $F_{Z^i}$ as follows:

$$\begin{aligned} F_{\textbf{Z}}(z_1,\ldots ,z_d) = C\left( F_{Z^1}(z_1),\ldots ,F_{Z^d}(z_d)\right) \end{aligned}$$

Then, the copula *C* can be used to draw dependent variates for each compound loss $Z^{i{\ast}}$, and subsequently the insurance premiums can be computed on the corresponding random vector of losses $X^{i{\ast}} = \left[ X_1^{i{\ast}},\ldots ,X_{N^{i{\ast}}}^{i{\ast}}\right]$. The steps of the algorithm in Cruz et al. (2015) are summarised in the following pseudocode.
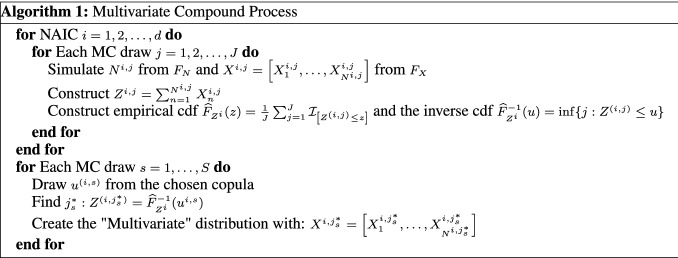


We will now be particularly interested in the effect on diversification of this insurance portfolio coming from different dependence estimates arising from various assumptions about the extreme losses in the Advisen cyber risk data and how they manifest in parameter uncertainty and model risk that we conjecture also translates into impact on an actuarie’s perspective of the diversification of the insurance portfolio.

When the loss distributions exhibit extreme dependence, such as comonotonicty, and extreme tail behaviour, even well diversified positions fail to produce diversification benefits (Wang and Dhaene 1998; Dahen and Dionne 2010; Ibragimov et al. 2011). According to Nešlehová et al. (2006), in the case of infinite mean distribution, the value at risk cannot be considered a coherent risk measure anymore, since subadditivity doesn’t hold. In particular, when independent Pareto-type heavy-tailed risk sources are pooled together, the resulting value at risk becomes superadditive:

33 $$\begin{aligned} {\text{VaR}}_{\alpha } \left( \sum _{i=1}^I Z^i\right) >\sum _{i=1}^I {\text{VaR}}_{\alpha }(Z^i). \end{aligned}$$

To show how this affects the diversification benefit we consider the standard diversification measure given by the ratio of the value at risk of the position, and the weighted average of the value at risk of each NAIC:

34 $$\begin{aligned} D(Z) = \frac{{\text{VaR}}_{\alpha } (\sum _{i=1}^I \omega _i Z^i) }{\sum _{i=1}^I \omega _i{\text{VaR}}_{\alpha }(Z^i)}. \end{aligned}$$

In ideal conditions, *D*(*Z*) is bounded in [0, 1], however we will show that this is not the case for cyber risk related losses in the Advisen data.

Table 6 shows quarterly and yearly premium and diversification values for a company holding an equally weighted position in the five NAICs and a total wealth of USD 10 billion dollars. For each robust correlation estimator, we allow for a Gaussian copula as the dependence structure and generate the random losses using the algorithm in Eq. 1.

**Table 6:** This table shows the insurance premiums and the diversification measure for an equally weighted portfolio of NAIC52, NAIC51, NAIC56, NAIC54 and NAIC92

Correlation estimator	Quarterly premium	Quarterly diversification	Yearly premium	Yearly diversification
Pearson correlation	2157.723	1.754	7647.262	1.406
	(59.014)	(0.191)	(43.951)	(0.268)
SSD median	2059.880	1.867	7482.660	1.269
	(59.540)	(0.295)	(44.215)	(0.215)
Quadrant (sign)	2018.442	1.547	7389.340	1.296
	(59.963)	(0.1883)	(43.069)	(0.177)
MCD	1989.372	1.643	7313.716	1.419
	(59.662)	(0.1910)	(43.833)	(0.194)

The estimator is reported along with the parameter standard error in brackets

Yearly and quarterly premiums in Table 6 can be rearranged into a decreasing order starting with those corresponding to liner Pearson correlation and followed by SSD, quadrant (sign) correlation, and MCD correlation estimators. Combining these results with the correlation estimates in Fig. 10, where robust correlation estimators give higher estimates than the linear Pearson correlation, it can be inferred that under the combination of heavy-tailed loss model marginals, combined with an elliptical copula with no tail dependence, such as the Gaussian copula model specification, then insurance premiums are negatively related with correlation estimates: ceteris paribas, as dependence strengthens, insurance premiums reduce.

Moreover, the variability among the various robust correlation estimates also translates into insurance premium uncertainty that can be interpreted as potential mispricing if the data records for extreme losses that drive these model risk and parameter uncertainties identified are not adequately accounted for in the pricing calculations. In such cases the resulting premiums are shown to vary according to the underlying assumptions about the data and model used in making an estimate of the dependence structure based on the correlations between the cyber risk loss processes.

One can also observe that the diversification measure returns values greater than 1, both on a quarterly and a yearly basis. Given that the portfolio evaluated in this case is formed by heavy-tailed risks, this is in agreement with the literature on diversification traps (Ibragimov et al. 2011). In instances where the underlying risks are heavy-tailed, the value at risk cannot be considered a subadditive risk measure any longer and therefore, the resulting diversification measure is not bounded in the interval [0, 1]. Moreover, the diversification measure defined in Eq. 34 is in general not consistent with majorisation orderings and in particular with the first order of stochastic dominance. Therefore, while insurance premiums are consistent with the riskiness of the position, this is not true in the case of the diversification measure. This is also confirmed by the values taken by the diversification measure in the various setting not following the ordering structure of the insurance premiums. Nevertheless we present these results for this measure of risk diversification as it is widely used in practice and so should be informative for practitioners.

The premiums in Table 6 reflect how the uncertainty in correlation estimates affect the net exposure of a portfolio of five sources of cyber risk. To evaluate how the dependence structure affects the exposure of each individual NAIC we consider performing a sequence of conditional premium calculations. For each NAIC and each correlation estimator, the insurance premiums are computed using the zero utility principle and solving the following non-linear equation modified to find the conditional cases:

35 $$\begin{aligned}& {\mathbb{E}}\left[ u\left( w-Z^i\right) \left| Z^s\ge F^{-1}_{Z^S}(u),s \ne i \right] \right.\\ & \quad \left. = {\mathbb{E}}\left[ u\left( w-P -Z^i+\sum _{n=1}^{N^i}\min (X_n^i,cw)\right) \right| Z^s\ge F^{-1}_{Z^S}(u),s \ne i \right] . \end{aligned}$$

We compute the conditional distribution for each case from the joint distribution generated by Algorithm 1. Table 7 shows the insurance premium computed on a quarterly basis, using the conditional equivalent principle of Eq. 35, for a representative company with USD 10 billion of wealth. The responsiveness of each NAIC to losses greater than 75% in the other business sectors can then be assessed in the subsequent results in Table 7.

**Table 7:** This table shows conditional insurance premiums and their bootstrapped standard errors, for the five NAICs

NAIC	Corr	SSD	Quadrant	MCD
NAIC52	7219.310	6607.928	6359.669	5820.162
	(175.775)	(176.543)	(162.363)	(163.646)
NAIC51	9521.399	9348.967	9202.159	8802.254
	(45.075)	(54.860)	(59.063)	(79.150)
NAIC56	5017.681	5189.146	4768.389	4982.309
	(213.505)	(195.435)	(189.904)	(184.620)
NAIC54	8692.571	8499.866	7889.639	7994.849
	(102.66)	(104.930)	(120.323)	(111.253)
NAIC92	2151.384	1971.347	1655.238	1617.179
	(211.624)	(183.206)	(154.234)	(147.890)

Each premium is computed assuming losses greater than 75% in the other NAICs, a company wealth of USD 10 billion, and the relevant correlation structure

The insurance premiums in Table 7 follow the same structure of the Hill estimates in Fig. 7, with NAIC51 reaching the highest values in all the considered dependence structures, suggesting that individual tail behaviour is still the main driver affecting insurance premium calculation, even when dependence structure is considered in the modelling. Comparing the premium sizes with Table 6 it can be seen that for each NAIC the highest conditional premium is achieved in the linear Pearson correlation case, while MCD and quadrant (sign) correlation return respectively the lowest estimates. Moreover, except in the case of NAIC92 based on MCD and quadrant (sign) correlation, conditional premiums are greater than net premiums, which is consistent with the increased risk of the positions analysed.

Insurance premiums from Tables 6 and 7 show the risk of a combination of NAICs and how pronounced the effect of parameter uncertainty on insurance pricing is. Nonetheless, the two approaches can be used in different situations. The portfolio approach can be used to quantify how exposed a company is on the cyber risk front, and how variation in positions could improve the risk profile. In this context, parameter uncertainty *externally* affects the enterprises under investigation, in the sense that among other things, the insurability of a company's cyber risk profile and stakeholder evaluation can be affected. The conditional calculation instead can be used *internally* to evaluate strategically which risk management and mitigation strategies are best suited for the given company's cyber risk profile. Here the effect of parameter uncertainty has the potential to be more catastrophic since it could ultimately lead to suboptimal or wrong decisions in the risk management department, or by the chief financial officer, where funds get misallocated to prevent or reduce the risk of catastrophic cyber events. Furthermore, there is a clear risk of mispricing insurance premiums associated with the misspecified risk profile of the insurance portfolio, which could result in loss of competition, customers or even regulatory scrutiny and fines.

### Dependence structures and copula

In this section we will undertake a copula dependence study, similar in nature to that explored in Eling and Jung (2018) in that we also explored pairwise dependence relationships. However, unlike this work, which explored data breach events from 2005 to 2016 in monthly quantisation bins in two cross-sectional settings: cross-industry losses in four categories by breach type (hacking, lost electronic device, unintended disclosure and insider breach) and cross-breach type losses in five categories by industry (banking and insurance, government, medical service, retail/other business and educational institution), we explore quarterly quantisation and compare dependence relationships across the pairs of NAIC sectors. Like Eling and Jung (2018) we also found evidence for significant asymmetric dependence of quarterly losses between NAIC business sectors.

In this last section we will explore the copula dependence structure for pairs of leading NAIC sectors. Fitting higher order copulas will be challenging due to the small sample sizes that arise from aggregating the loss data to a three-monthly stratification. Recall, this period was selected to ensure reasonable sample sizes over time, so we have therefore intentionally restricted to two dimensional copula analysis as a result. Nevertheless this is still an insightful analysis to perform. Here we focus on the impact of selecting the *right* dependence structure on insurance pricing and diversification measure. Given the quality and quantity of the data, instead of finding the best copula that fits the five NAICs jointly, we resort to variational approximation, where the true distribution is approximated by the combination of independent pair copulas that minimises the Kullback–Leibler divergence. We proceed with the following steps:

- *Step 1* we identify the best copula for each NAIC pair according to an information criterion;
- *Step 2* fit independence copula for each combination of pair copulas where marginals appear uniquely;
- *Step 3* select the combination of independent pair copulas that minimises the Kullback–Leibler divergence.

Table 8 shows the results of the copula selection procedure according to the Akaike Information Criterion, the corresponding copula parameter estimated using maximum likelihood, and Kendall’s $\tau$. As shown in in the table, there appears to be not much variation in terms of the selected copula, copula parameter and Kendall’s $\tau$ since the Joe copula is systematically selected as the best choice for each pair, and the parameters do not vary much between this model for each pair. This seems to suggest that when taking into account tail dependence, all five NAICs have very similar behaviour, showing a positive tail dependence.

**Table 8:** Best copula, and corresponding estimated parameter $\theta$ and Kendall’s $\tau$ selected by the package VineCopula using Akaike information criterion

	NAIC52	NAIC51	NAIC56	NAIC54	NAIC92
NAIC52	–	Survival Joe	Survival Joe	Survival Joe	Survival Joe
	–	$\widehat{\theta } = 4.19$, $\widehat{\tau }= 0.63$	$\widehat{\theta } = 2.96$, $\widehat{\tau }= 0.51$	$\widehat{\theta } =4.09$, $\widehat{\tau }= 0.62$	$\widehat{\theta } =3.33$, $\widehat{\tau }= 0.55$
NAIC51	–	–	Survival Joe	Survival Joe	Survival Joe
	–	–	$\widehat{\theta } = 3.85 , \widehat{\tau } = 0.6$	$\widehat{\theta } = 4.7, \widehat{\tau } = 0.66$	$\widehat{\theta } =3.58 , \widehat{\tau } = 0.58$
NAIC56	–	–	–	Survival Joe	Survival Joe
	–	–	–	$\widehat{\theta } = 3.47, \widehat{\tau } =0.57$	$\widehat{\theta } =3.11 , \widehat{\tau } = 0.53$
NAIC54	–	–	–	–	Survival Joe
	–	–	–	–	$\widehat{\theta } = 3.17 , \widehat{\tau } = 0.54$
NAIC92	–	–	–	–	–
	–	–	–	–	–

**Fig. 11:**
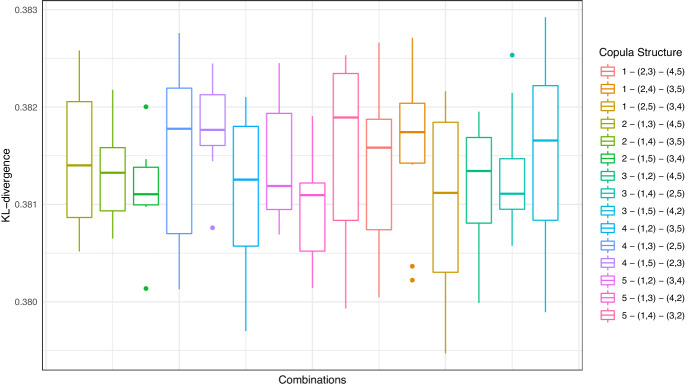
KL divergence of different copula structure ordered by tail parameter of the independent component for different seed. The values of KL divergence remain similar for different copula structure combinations. The legend reads as follows: 1: NAIC52, 2: NAIC51, 3:NAIC56, 4: NAIC54, 5: NAIC92

Given that our analysis focused on an odd number of NAICs, we form the combined five dimensional model as comprised of a product of two dimensional copulas in the variational approximation keeping one component independent, while allowing pair copulas for the other four NAICs. Figure 11 shows the Kullback–Leibler divergence between all the possible combinations of pair copulas and independent component for different seed in the random number generator, ordered according to the tail index estimates of the independent component. As can be seen, the relatively flat structure in Table 8 affects also the KL divergence results, where the values are so close to each other that even the small and almost negligible variation due to the random number generation could affect the results.

**Fig. 12:**
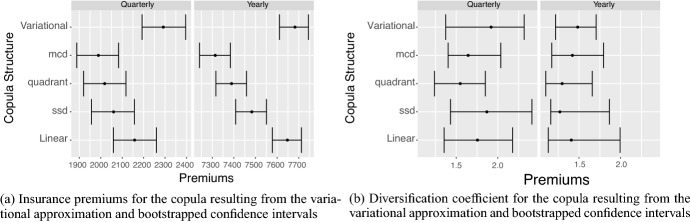
Insurance premiums based on log utility and diversification measure of an equally weighted portfolio of five NAICs, with bootstrapped 95% confidence intervals

**Fig. 13:**
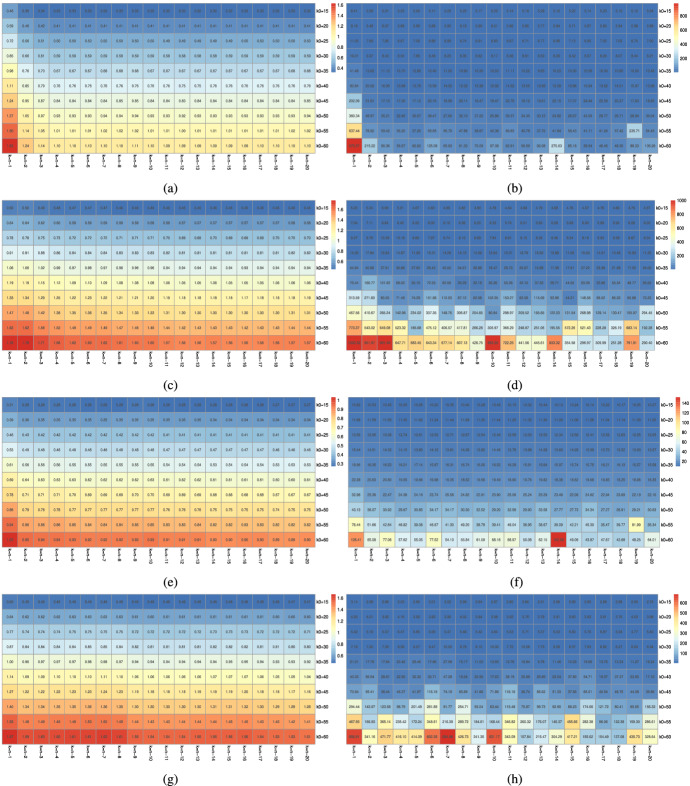
Trimmed inverse tail index Hill estimators (left column) and corresponding insurance premiums (right column) for varying trimming parameters $(k_0,k)$ for **a** NAIC51, **b** NAIC54, **c** NAIC56, **d** NAIC92. Premiums are computed on a quarterly basis using 1000,000 Monte Carlo draws. The variation in the trimmed Hill estimates translates into premium calculations

Since selecting a best performing approximated copula structure is not possible, we present the results for the case where NAIC51 is left as an independent component, NAIC52 and NAIC54 are fitted on a Joe copula with parameter $\theta = 4.09$, and NAIC56 and NAIC92 are fitted on a Joe copula with parameter $\theta = 3.11$. Similar results can be obtained with other combinations of independent component and pair copulas. Then we use the selected copula structure as a basis for the simulation in Algorithm 1 and compute the insurance premium of Eq. 32 in the case of an equally weighted portfolio of NAICs, and the corresponding diversification measure. Figure 12 compares the results with those previously obtained using the Gaussian settings and the different robust correlation estimators, reporting also the bootstrapped 95% confidence intervals. Insurance premiums computed using the approximated copula are statistically different from those computed using the Gaussian copula with the robust correlation estimators, both on a quarterly and yearly basis. This indicates that not only parameter uncertainty affects insurance premium calculation in the case of cyber risk, but model risk does as well. Nevertheless, premiums based on the linear correlation estimator are not statistically different from the one computed using the approximated copula. This can be explained due to the presence of two conflicting biases. On the one hand, the assumption of a Gaussian copula as a joint dependence structure seem to increase the values of insurance premiums. On the other hand, robust correlation estimators reduce the premium values, resulting in a premium not statistically different than the one computed using the approximated copula structure. Looking at the diversification measure results, there appear not to be any statistically significant differences between the considered underlying dependence structures: the diversification measure fails to be bounded in the interval [0, 1] due to the lack of subadditivity, and seems to have a more skewed bootstrapped distribution with respect to the insurance premiums, having the mean not centred in the confidence intervals. Finally, it can be noticed how the confidence intervals for the diversification measure seem to be less affected by time aggregation than the premium counterparts. This can be explained by the lack of subadditivity due to the heavy tails of the considered risks: in the bootstrap procedure it’s more likely that extreme scenarios, violating the subadditivity, are generated more often.

Figure 12 provides statistical evidence that model risk and parameter uncertainty in cyber risk translate into insurance premiums and could affect the decision making process.

## Conclusions

The paper explored the relationship between model risk and parameter uncertainty in insurance pricing in the setting of cyber risk. In particular the paper sought to explore whether the perspective previously held in the literature, that cyber risk losses are heavy-tailed, was consistently found in the largest industry standard loss database, obtained from Advisen. In this context the paper showed that ones perspective on the tail behaviour of cyber risk loss processes is heavily dependent on the ability to rely upon the properties of the data obtained for calibration. Given that in the industry leading database there was evidence of some of the largest losses being incompletely reported, rounded, approximated and never settled or realised, we decided to assess what impact this may have on the actuary's perspective of the tail behaviour of such cyber risk loss processes. This is particularly compounded by the fact that, necessarily so, the total cyber loss per event in the Advisen dataset is a composition of many direct and indirect loss components, where direct losses are from the event itself and indirect losses are from the consequences of the event. When the extremes of the data are contaminated, the classical Hill-type estimators lead to inaccuracy in utility-based cyber insurance premium calculations. Furthermore, it poses a challenge to assess the insurability of cyber risk losses.

Robust estimators were adopted rather than the standard tail index estimators that used all data as equally weighted and applied no trimming. To improve the robustness of the tail index estimator reducing the effect of such observations (i.e. extreme outliers), we used the trimmed Hill estimator on aggregated five NAICs cyber-related losses. We noticed that while it is highly sensitive to the choice of trimming parameters for each NAIC, the uncertainty of the trimmed Hill estimator ultimately affects premium mispricing. Consequently, model risk makes it difficult to assess the insurability of cyber risk losses. This led to the conclusion that significant model risk and parameter uncertainty may be present in the analysis depending on ones perspective on assessing the quality of the real data. Furthermore, we showed that once this was translated into insurance pricing, this led to significant mispricing potential in charged premiums.

We also investigated how uncertainty of the dependence structure of cyber event severity between five NAICs impacted on utility-based conditional/unconditional cyber insurance premium pricing and diversification benefit measures. Dependence between five NAICs was studied via robust dependence estimation methods, copula estimation methods, and a little known Monte Carlo based simulation method and the value-at-risk ratio was used as the diversification measure.

We provided statistical evidence that cyber premium mispricing and misleading diversification benefit measuring can arise from dependence model structure uncertainty as well as relevant parameter uncertainty. We hope that what we have presented in this paper will provide practitioners with sensible approaches to quantify/assess univariate/multivariate cyber losses over a range of different industry sectors dealing with model risk.

## Appendix 1

See Fig. 13.
